# Autonomous emergence of connectivity assemblies via spike triplet interactions

**DOI:** 10.1371/journal.pcbi.1007835

**Published:** 2020-05-08

**Authors:** Lisandro Montangie, Christoph Miehl, Julijana Gjorgjieva

**Affiliations:** 1 Computation in Neural Circuits Group, Max Planck Institute for Brain Research, Frankfurt, Germany; 2 Technical University of Munich, School of Life Sciences, Freising, Germany; University of Pittsburgh, UNITED STATES

## Abstract

Non-random connectivity can emerge without structured external input driven by activity-dependent mechanisms of synaptic plasticity based on precise spiking patterns. Here we analyze the emergence of global structures in recurrent networks based on a triplet model of spike timing dependent plasticity (STDP), which depends on the interactions of three precisely-timed spikes, and can describe plasticity experiments with varying spike frequency better than the classical pair-based STDP rule. We derive synaptic changes arising from correlations up to third-order and describe them as the sum of structural motifs, which determine how any spike in the network influences a given synaptic connection through possible connectivity paths. This motif expansion framework reveals novel structural motifs under the triplet STDP rule, which support the formation of bidirectional connections and ultimately the spontaneous emergence of global network structure in the form of self-connected groups of neurons, or assemblies. We propose that under triplet STDP assembly structure can emerge without the need for externally patterned inputs or assuming a symmetric pair-based STDP rule common in previous studies. The emergence of non-random network structure under triplet STDP occurs through internally-generated higher-order correlations, which are ubiquitous in natural stimuli and neuronal spiking activity, and important for coding. We further demonstrate how neuromodulatory mechanisms that modulate the shape of the triplet STDP rule or the synaptic transmission function differentially promote structural motifs underlying the emergence of assemblies, and quantify the differences using graph theoretic measures.

## Author summary

Emergent non-random connectivity structures in different brain regions are tightly related to specific patterns of neural activity and support diverse brain functions. For instance, self-connected groups of neurons, known as assemblies, have been proposed to represent functional units in brain circuits and can emerge even without patterned external instruction. Here we investigate the emergence of non-random connectivity in recurrent networks using a particular plasticity rule, triplet STDP, which relies on the interaction of spike triplets and can capture higher-order statistical dependencies in neural activity. We derive the evolution of the synaptic strengths in the network and explore the conditions for the self-organization of connectivity into assemblies. We demonstrate key differences of the triplet STDP rule compared to the classical pair-based rule in terms of how assemblies are formed, including the realistic asymmetric shape and influence of novel connectivity motifs on network plasticity driven by higher-order correlations. Assembly formation depends on the specific shape of the STDP window and synaptic transmission function, pointing towards an important role of neuromodulatory signals on formation of intrinsically generated assemblies.

## Introduction

The synaptic wiring between neurons—originally proposed as a mechanism for learning and memory—is sculpted by experience and has become a most relevant link between circuit structure and function [1]. The original formulation of Hebbian plasticity, whereby “cells that fire together, wire together” [2, 3], fostered the concept of ‘cell assemblies’ [4], defined as groups of neurons that are repeatedly co-activated leading to the strengthening of synaptic connectivity between individual neurons. This has suggested that activity-dependent synaptic plasticity, including both long-term potentiation and long-term depression, is a key mechanism for the emergence of assemblies in the organization of neural circuits [5–7]. These interconnected groups of neurons have become an important target for many theories of neural computation and associative memory [8–11]. Recent technological developments that enable multiple neurons to be simultaneously recorded have provided the much needed physiological evidence of assembly organization [12–15]. For instance, synaptically connected neurons tend to receive more common input than would be expected by chance, [12, 16–18] and cortical pyramidal neurons tend to be more strongly connected to neurons that share stimulus preference [13, 19, 20], providing evidence for clustered architecture. It has been proposed that this organization enables the cortex to intrinsically generate reverberating patterns of neural activity when representing different stimulus features [1, 21]. Thus, neuronal assemblies can be interpreted as the building blocks of cortical microcircuits which are differentially recruited during distinct functions, such as the binding of different features of a sensory stimulus [7, 17, 22]. In addition to cortical circuits, neuronal assemblies have also been observed in the optic tectum (a structure homologous to the superior colliculus in mammals [23]) in the developing zebrafish larva [24–27]. Experiments in sensory deprived larvae have demonstrated that the basic structure of spontaneous activity and functional connectivity emerges without intact retinal inputs, suggesting that neuronal assemblies are intrinsically generated in the tectum and not just the product of correlated external inputs [25–27]. This raises the important question of what drives the emergence of these clustered structures, and whether patterned external input is necessary.

To understand the emergence of such non-random connectivity, a growing body of theoretical and computational work has been developed to link connectivity architecture to the coordinated spiking activity of neurons, especially in recurrent networks [28–41]. These studies can be divided into two classes: those that examine the influence of externally structured input on activity-dependent refinement [42–47], and those that investigate the autonomous emergence of non-random connectivity in the absence of patterned external input, purely driven by emergent network interactions [5, 6, 48]. Specifically, assemblies in recurrent networks can be imprinted based on internally-generated network interactions [6] or through rate-based plasticity where inputs with higher firing rates to subsets of neurons strengthen recurrent connections [49, 50]; assemblies can also be initially determined by externally patterned input but maintained by internal correlations [51].

Despite this success, all of these studies have assumed pair-based models of STDP, which induce plasticity based on the precise timing and order of a pair of pre- and postsynaptic spikes [52, 53]. Here, we consider a spike-based learning rule, “the triplet STDP model” [54], which uses sets of three spikes (triplets) to induce plasticity. Specifically, we focus on the ‘minimal’ triplet STDP model, where only potentiation depends on the interval between the pre- and postsynaptic spikes, and on the timing of the previous postsynaptic spike. This triplet learning rule has been shown to explain a variety of synaptic plasticity data [55, 56] significantly better than pair-based STDP [54]. We have previously shown a tight correspondence between the triplet STDP rule and the well-known Bienenstock-Cooper-Munro (BCM) synaptic learning rule, which maximizes the selectivity of the postsynaptic neuron, and thereby offers a possible explanation for experience-dependent cortical plasticity such as orientation and direction selectivity [57]. In addition, triplet STDP can also induce selectivity for input patterns consisting of up to third-order correlations, here referred to as higher-order correlations (HOCs). HOCs have been experimentally measured in several brain areas [58], and shown to account for a substantial amount of information transfer in sensory cortex [58–61]. HOCs are also important for characterizing the firing of a postsynaptic neuron [62, 63], circuit function and coding [64, 65], and the synchronous firing and the distribution of activity in a neuronal pool [66–69]. Here we investigated the functional significance of such HOCs for shaping recurrent network structure through synaptic plasticity.

First, we investigate how HOCs up to third order affect the development of connectivity in recurrent networks of Poisson spiking neurons in the absence of structured external stimuli, where the stochastic activity of each neuron is described by a mutually exciting Hawkes process [70]. Assuming a slow change of synaptic efficacies and fast spiking dynamics, we develop a formal analytical framework for the evolution of synaptic connections in the network based on the second- and third-order cumulants of spike timing interactions, which arise from assuming an STDP rule governed by pairs and triplets of spikes [54, 55]. The simplified neuronal model allows us to write exact and self-consistent equations for the synaptic change depending on the full network connectivity by taking into account non-local interactions between different neurons in the network and writing them as a sum of structural motifs of varying orders. We demonstrate differences to the classical pair-based STDP rule [52, 71] that ignores those HOCs, and compare the relative strength of the emergent structural motifs up to third-order induced by triplet STDP. Second, we examine the biological conditions which promote the formation of assembly structures of self-connected neurons without externally structured inputs under the triplet STDP rule. We find that this is achieved either by modulating the shape of the STDP function through neuromodulators or the shape of the evoked postsynaptic current (EPSC) and characterize changes in functional connectivity in terms of graph theoretic measures [25–27]. Third, we show that the novel structural motifs, and specifically ‘loop’ motifs, which follow from the triplet STDP rule, are crucial for the spontaneous emergence of assemblies. Finally, we compare them to assemblies generated via correlated external input.

## Results

We present two main results: first, we derive a formal analytical framework for the evolution of synaptic weights depending on the second- and third-order cumulants of spike time interactions under the triplet STDP rule by expressing them as a sum of structural motifs; second, we discuss the functional implications of this framework and present the biological conditions which promote the formation of assemblies without external instruction.

### Average synaptic modification due to the interaction of pairs and triplets of spikes in recurrent networks

To study the autonomous emergence of assemblies in a recurrent network from a general form of STDP that includes the contribution of pairs and triplets of spikes to synaptic plasticity, we require a minimal theoretical representation of the network with plastic synapses driven by internal correlations in the spike timing statistics. In our model, structure is given by the connectivity matrix ***W*** between all excitatory neurons in the network (“all-to-all connectivity”), where the synaptic efficacy element *W*_*ij*_ denotes the connection strength between postsynaptic neuron *i* and presynaptic neuron *j*. The analytical description of the dynamics in recurrent networks can be dauntingly complex. On the one hand, to rigorously analyze the impact of STDP on the formation of functional structures it is indispensable to take into account the precise timing of action potentials or spikes. Therefore, models of neural activity that are based on rates cannot fulfill this criterion [72]. More elaborate models such as Hodgkin-Huxley with multiple ion channels [73] and even the simpler spiking leaky integrate-and-fire (LIF) models are much more accurate in reproducing the spiking dynamics of a population of neurons [74–76]. Although they are computationally tractable, to extract extensive and exact mathematical features from these models remains an elusive task. Under certain conditions of approximately asynchronous firing, the spiking statistics in networks of LIF neuron can be described by a linear theory [29]. Using this approach, here we make approximations for the spiking dynamics of each individual excitatory cell and treat each pre- and postsynaptic spike train as if they follow inhomogeneous Poisson statistics [6, 44, 52, 57].

**Fig 1:**
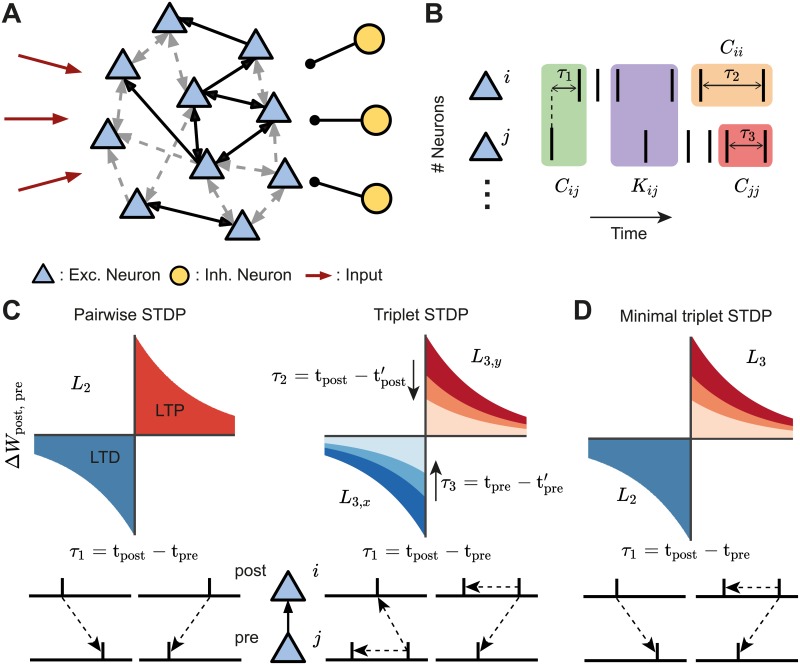
Framework set-up. **A**. A network of excitatory neurons (light blue triangles) fire stochastically, while their activity is driven by unstructured external input (red arrows) and modulated by a population of inhibitory neurons (yellow circles). Excitatory connections among the neurons can be weak (gray dashed arrows) or strong (black solid arrows), unidirectional or bidirectional. **B**. Cumulants of the spike trains (see Eq 1). The second-order cumulants *C*_*ij*_, *C*_*ii*_ and *C*_*jj*_ are calculated based on the time difference between a pair of spikes (cross-covariance in green; auto-covariances in orange/red). The third-order cumulant *K*_*ij*_ is calculated based on the time differences between three spikes (purple). The spike triplets can be two post- and one presynaptic spikes, or one post- and two presynaptic spikes. The time differences are: *τ*_1_ between a presynaptic spike and a postsynaptic spike, *τ*_2_ between different postsynaptic spikes and *τ*_3_ between different presynaptic spikes. **C**. STDP-induced plasticity by pairs and triplets of spikes. Left: An example of a classical pair-based STDP rule, with a learning window denoted by *L*_2_. Potentiation is triggered by a postsynaptic following a presynaptic spike (*τ*_1_ = t_post_ − t_pre_ > 0), whereas if a presynaptic spike follows a postsynaptic spike (*τ*_1_ = t_post_ − t_pre_ < 0), depression is induced. The total potentiation (depression) is given by the red (blue) area under the curve. Right: Examples of triplet STDP rules denoted by *L*_3,*y*_ and *L*_3,*x*_. Potentiation (red) and depression (blue) are given by triplets of spikes: post-pre-post with a time difference $\tau_{2}={\text{t}}_{\text{post}}-{\text{t}}_{\text{post}}^{\prime }$, and pre-post-pre with a time difference $\tau_{3}={\text{t}}_{\text{pre}}-{\text{t}}_{\text{pre}}^{\prime }$, respectively. **D**. Minimal triplet STDP rule where potentiation depends on triplets of spikes *L*_3_ and depression on pairs of spikes *L*_2_.

In this model we assume that the probability of each neuron emitting an action potential at a certain time (the ‘intensity’ or mean activity) is proportional to the weighted sum of the preceding activity of all the other cells in the network and a constant, unstructured external input (Fig 1A). The activity of each neuron in this network is a stochastic process, also referred to as a ‘mutually exciting point process’ or a Hawkes process [70]. The availability of an exact expression for spike correlations in this model allows us to develop a precise theory for the synaptic efficacies’ dynamics that are governed by different forms of STDP. To prevent runaway excitation, we also consider that the firing of excitatory neurons is modulated by the activity of a population of inhibitory neurons (Fig 1A). We assume that the total inhibitory input to each excitatory neuron is tuned in order to balance the sum of inhibitory efficacies with the sum of the excitatory ones (Methods) [6, 77–79].

Given the connectivity matrix ***W*** and assuming a slow learning rate (much slower than the dynamics of neural activity), the rate of change in the strength of synaptic efficacy $\langle {\dot{W}}_{ij}\rangle$ between postsynaptic neuron *i* and presynaptic neuron *j*, can be expressed in terms of the product of the time dependent cumulants of different orders and the STDP function, accordingly (Methods). Specifically, we consider STDP learning rules where plasticity depends on the timing and order of pairs and triplets of spikes, referred to as pair-based and triplet STDP. Initially, we make no assumptions about the shape of these learning rules keeping the framework general. The sign and magnitude of the net weight modification depends on the time interval between the firing of the pre- and postsynaptic neurons, and also on the relative spike times of individual pre- and postsynaptic neurons (Fig 1B). The exact expression for the evolution of the average (denoted by 〈⋅〉) synaptic efficacy in the recurrent network due to STDP is

$$\begin{array}{c} \begin{array}{cc} & \left\langle {\dot{W}}_{ij}^{\text{STDP}}\right\rangle =\int_{-\infty }^{\infty }(C_{ij}\left(\tau_{1}\right)+r_{i}r_{j})L_{2}\left(\tau_{1}\right)\text{d}\tau_{1}\\ & +\overset{\infty }{\underset{-\infty }{\;\int \int \;}}(K_{ij}\left(\tau_{1},\tau_{2}\right)+r_{i}(C_{ij}\left(\tau_{1}\right)+C_{ij}\left(\tau_{2}-\tau_{1}\right))+r_{j}C_{ii}\left(\tau_{2}\right)+r_{i}^{2}r_{j})L_{3,y}\left(\tau_{1},\tau_{2}\right)\text{d}\tau_{1}\;\text{d}\tau_{2}\\ & +\overset{\infty }{\underset{-\infty }{\;\int \int \;}}(K_{ij}\left(\tau_{1},\tau_{3}\right)+r_{j}(C_{ij}\left(\tau_{1}\right)+C_{ij}\left(\tau_{3}-\tau_{1}\right))+r_{i}C_{jj}\left(\tau_{3}\right)+r_{i}r_{j}^{2})L_{3,x}\left(-\tau_{1},-\tau_{3}\right)\text{d}\tau_{1}\;\text{d}\tau_{3}. \end{array} \end{array}$$ (1)

Here *r*_*i*_ and *r*_*j*_ denote the mean firing rates of neuron *i* and *j*, respectively; *C*_*ij*_ is the cross-covariance between neuron *i* and neuron *j*, with *C*_*ii*_ and *C*_*jj*_ being the auto-covariances (note that all of these covariance terms, *C*_*ij*_, *C*_*ii*_ and *C*_*jj*_, make up the second-order cumulant); and *K*_*ij*_ is the third-order cumulant between neuron *i* and neuron *j*. These quantities represent internal (i.e. not driven by external input) correlations in the network and are calculated as functions of the excitatory postsynaptic current (EPSC), and assumed to be identical for every pair of neurons. Both the second-order cumulants *C* and the third-order cumulants *K* are probability densities of pairs and triplets of spikes separated by the given time lapses *τ* accordingly (Fig 1B). *τ*_1_ is the time difference between a spike emitted by the presynaptic neuron and one from the postsynaptic neuron, whereas *τ*_2_ and *τ*_3_ are the time intervals between different spikes from the postsynaptic neuron and the presynaptic neuron, respectively. The cumulant *K*_*ij*_ is calculated for both ‘post-pre-post’ or ‘pre-post-pre’ spike triplets and therefore depends on combinations of *τ*_1_ and *τ*_2_ or *τ*_3_, according to each case.

The STDP functions that describe how potentiation or depression depend on the spike timing intervals are given by *L*_2_ for pairs of spikes, and *L*_3,*x*_ and *L*_3,*y*_ for triplets of spikes. The sub-indices *x* and *y* correspond to the triplet sets ‘pre-post-pre’ and ‘post-pre-post,’ respectively. While Eq 1 can be calculated for any shape of the STDP function that depends on pairs and triplets of spikes, an illustrative example for these learning rules, commonly used in other studies based on fits to experimental data [54, 55, 71], is given in Fig 1C.

The average synaptic efficacy change (Eq 1) is sufficient to describe the plasticity dynamics when the learning rate is small relative to the spiking dynamics, and noise in the STDP dynamics, arising from random fluctuations, is averaged out. Furthermore, Eq 1 is combined with heterosynaptic competition [80] to restrict the amount of connections a neuron can make with the rest and prevent the dominance of a few (Methods). For the sake of simplicity, in the next steps we consider that triplets of spikes contribute only to potentiation and thus *L*_3,*y*_(*τ*_1_, *τ*_2_) = *L*_3_(*τ*_1_, *τ*_2_) and *L*_3,*x*_(*τ*_1_, *τ*_3_) = 0, for all *τ*_1_ and *τ*_3_, in agreement with the so-called ‘minimal’ triplet STDP rule [54] (Fig 1D). Nevertheless, if spike triplets would also be taken into account for depression, the derivation would be identical, with the corresponding modification to the variables involved. We can rewrite Eq (1) in the Fourier domain as

$$\begin{array}{c} \begin{array}{cc} \left\langle {\dot{W}}_{ij}^{\text{STDP}}\right\rangle & =\underset{\text{Independent}\;\text{spikes}\;}{\underset{︸}{r_{i}r_{j}({\tilde{L}}_{2}\left(0\right)+r_{i}{\tilde{L}}_{3}\left(0,0\right))}}\\ & +\underset{\text{Pre-post}\;\text{pairwise}\;\text{correlations}\;(*)}{\underset{︸}{\overset{\infty }{\underset{-\infty }{\;\int \int \;}}[{\tilde{C}}_{ij}\left(\omega_{1}\right){\tilde{L}}_{2}\left(-\omega_{1}\right)\delta \left(\omega_{2}\right)+r_{i}({\tilde{C}}_{ij}\left(\omega_{1}\right)\delta \left(\omega_{2}\right)+{\tilde{C}}_{ij}\left(\omega_{2}\right)\delta \left(\omega_{1}+\omega_{2}\right)){\tilde{L}}_{3}\left(-\omega_{1},-\omega_{2}\right)]\text{d}\omega_{1}\;\text{d}\omega_{2}}}\\ & +r_{j}\underset{\text{Post-post}\;\text{pairwise}\;\text{correlations}\;(**)}{\underset{︸}{\overset{\infty }{\underset{-\infty }{\;\int \int \;}}{\tilde{C}}_{ii}\left(\omega_{2}\right){\tilde{L}}_{3}\left(-\omega_{1},-\omega_{2}\right)\delta \left(\omega_{1}\right)d\omega_{1}d\omega_{2}}}+\underset{\text{Post-pre-post}\;\text{triplet}\;\text{correlations}\;(***)}{\underset{︸}{\overset{\infty }{\underset{-\infty }{\;\int \int \;}}{\tilde{K}}_{ij}\left(\omega_{1},\omega_{2}\right){\tilde{L}}_{3}\left(-\omega_{1},-\omega_{2}\right)\text{d}\omega_{1}\;\text{d}\omega_{2}}} \end{array} \end{array}$$ (2)

where we use the notation $\tilde{f}$ for the Fourier transform of a function *f* and *δ* is the Dirac delta function. It should be noted that Eq 2 is not the Fourier transform of Eq 1 but rather an equivalent expression of the latter. This comes about because we can express the integral of the product of two functions as the convolution of the Fourier transform of those functions, evaluated at zero.

This formulation of the previous equation allows us to clearly break down the contribution of spike interactions of different orders to the average synaptic efficacy in the recurrent network. The first term of Eq 2 considers the change in synaptic efficacy that is obtained from independent spiking and thus depends on the first-order cumulant (the mean firing rates) of the activity of both the pre- and postsynaptic neurons, *r*_*j*_ and *r*_*i*_, respectively. As firing rates increase, ‘chance’ contributions to plasticity can occur. The second and third term account for the probability of observing changes to the mean synaptic efficacy due to pairwise correlations in the pre- and postsynaptic neurons. *C*_*ij*_ refers to the family of probabilities that generate pairwise cross-correlations (second-order cumulant) between neurons *i* and *j*, depending on spikes of other neurons in the network (Fig 1B, green). Accordingly, *C*_*ii*_ includes the family of probabilities that generate pairwise auto-correlations in the same neuron *i* due to the spiking activity of all other neurons in the network (Fig 1B, orange). Therefore, the second (*) and third (**) terms describe the total contribution of correlated spike pairs to plasticity through the pair-based STDP rule *L*_2_ (Fig 1C, left) and the triplet STDP rule *L*_3_ (Fig 1C, right). In the case of the latter, the first-order cumulant of the uncorrelated single postsynaptic neuron’s spikes, *r*_*i*_, is also included in the second term (*) and the first-order cumulant of the uncorrelated single presynaptic neuron’s spikes, *r*_*j*_, in the third term (**). The fourth term (***) describes the total contribution of correlated spike triplets (third-order cumulant) to plasticity. Thus, *K*_*ij*_ includes the family of probabilities for third-order correlations, where the relative spike timing interacts with the triplet STDP learning window *L*_3_ to induce plasticity (Fig 1B, purple and Fig 1C, right).

In conclusion, we have derived an exact analytical expression for the average change in synaptic efficacy due to firing rates, pairwise and triplet correlations under a general STDP rule that includes pairwise and triplet spike interactions. The resulting cumulants of up to third order can depend in non-trivial ways on the full recurrent connectivity in the network.

### Novel structural motifs emerge under triplet STDP compared to pair-based STDP

**Fig 2:**
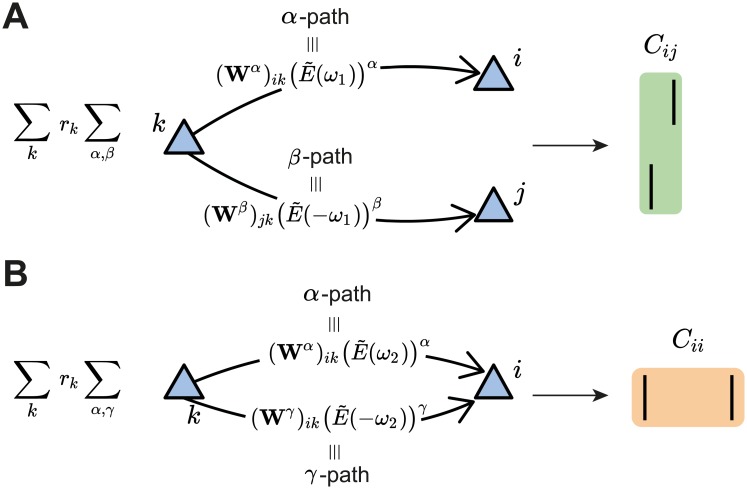
Second-order cumulant contributions to plasticity. **A**. The cross-covariance *C*_*ij*_ between the presynaptic neuron *j* and the postsynaptic neuron *i* is obtained by summing over all the possible *α*- and *β*-paths from every possible source neuron *k* in the network. Each path is calculated via the corresponding weights in the connectivity matrix and the EPSC function (see Eq 3). **B**. Same as **A** but for the auto-covariance *C*_*ii*_ of the postsynaptic neuron *i* (see Eq 4). In this case, *γ* is the second index to sum over the path from the source neuron *k* to the postsynaptic neuron *i*. It should be noted that the main difference between the *α*- and *γ*-path is given by the time dependence of the EPSC function, here represented in the Fourier domain for convenience.

The calculation of the cumulants involved in the equation for the average weight dynamics (Eq 2) depends on the full network connectivity. Therefore, the second- and third-order cumulants in Eq 2 can be written as a sum over contributions from different structural motifs, following the convention of [6]. These structural motifs determine all possible connectivity paths that a given spike from a source neuron *k* travels to the postsynaptic neuron *i* or presynaptic neuron *j*, and as a consequence affects the synaptic weight *W*_*ij*_. Thus, to calculate each term in Eq 2 we break down the second- and third-order cumulants *C*_*ij*_, *C*_*ii*_ and *K*_*ij*_ into expressions that include the contribution of every spike propagated in the network through existing synaptic connections, taking into account the full recurrence in the network (Methods). These expressions consist of products of the corresponding synaptic efficacies along the two paths to the presynaptic and postsynaptic neuron, the firing rate of the source neuron and the motif coefficient functions *M*, which depend on the number of synapses along the two paths, the EPSC function, *E*, and the STDP learning rules, *L*_2_ and *L*_3_. The probability that neurons *i* and *j* jointly fire a spike is transiently modulated whenever a neuron anywhere in the network produces a spike. We can write the pairwise cross-covariance from Eq 2 as

$$\begin{array}{c} \begin{array}{cc} \left(*\right) & \equiv \sum_{k=1}^{N}r_{k}\sum_{\alpha ,\beta }{\left(W^{\alpha }\right)}_{ik}{\left(W^{\beta }\right)}_{jk}(M_{\alpha ,\beta }^{pair}+r_{i}M_{\alpha ,\beta }^{trip}) \end{array} \end{array}$$ (3)

which combines the contribution of structural motifs from the pair-based and triplet STDP rules to a change in the connectivity matrix ***W***. The expression consists of sums over two aspects to provide an intuitive description of the contribution of the pairwise cross-covariance *C*_*ij*_ between neurons *i* and *j* to plasticity of the connection *W*_*ij*_. The first sum takes into account all spiking neurons in the network, while the second sum takes into account all possible ‘paths’ by which spikes originating from a ‘source’ neuron *k* affect the cross-covariance *C*_*ij*_. Specifically, *α* and *β* constitute the ‘path lengths’ of synapses from source neuron *k* to the postsynaptic neuron *i* and the presynaptic neuron *j*, respectively (Fig 2A; see also [6]). We refer to the total path length of a motif, *α* + *β*, as the ‘order’ of the motif.

**Fig 3:**
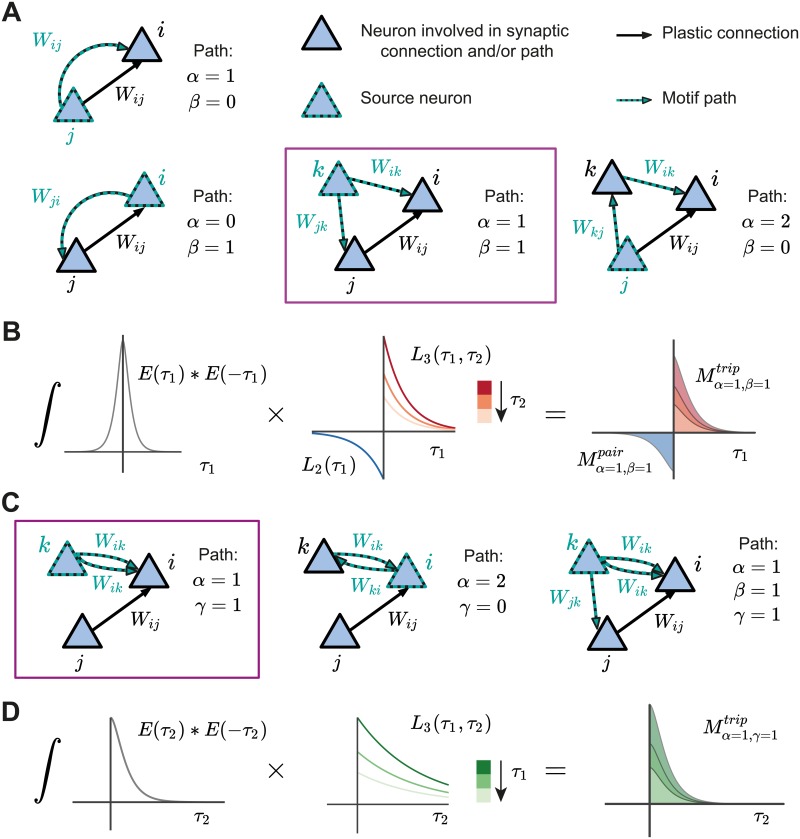
Structural motifs in the network under pair-based and triplet STDP. **A**. Examples of structural motifs common for both the pair-based and triplet STDP framework. Here *α* and *β* constitute the path lengths of synapses from the source neuron to the postsynaptic neuron *i* and the presynaptic neuron *j*. *α* = 1, *β* = 0: Presynaptic neuron *j* projects to the postsynaptic neuron *i*. *α* = 0, *β* = 1: Postsynaptic neuron *i* projects to the presynaptic neuron *j*. *α* = 1, *β* = 1: Common input from source neuron *k* to presynaptic neuron *j* and postsynaptic neuron *i*. *α* = 2, *β* = 0: Presynaptic neuron *j* projects to the postsynaptic neuron *i* through another neuron *k* in the network. **B**. Illustration of the calculation of the common input motif with *α* = 1 and *β* = 1 framed in purple in **A** (there are also additional terms which are not illustrated). The motif coefficients *M*_*α*=1,*β*=1_ (right) are calculated as the total area under the curve resulting from the product of the convolution of the EPSC function *E* (left) and the STDP functions (pair-based *L*_2_ and triplet *L*_3_, middle). **C**. Examples of structural motifs found only in the triplet STDP framework, where *γ* denotes the time-delayed path length from the source neuron to the postsynaptic neuron *i*. *α* = 1, *γ* = 1: Source neuron *k* projects twice to postsynaptic neuron *i* with a different time delay. *α* = 2, *γ* = 0: Feedback loop through another neuron *k* in the network (source and projecting neuron are the postsynaptic neuron *i*). *α* = 1, *β* = 1, *γ* = 1: Source neuron *k* projects to the presynaptic neuron *j* and postsynaptic neuron *i* via all the three possible paths. **D**. Illustration of the calculation of the motif with *α* = 1 and *γ* = 1 for the triplet STDP rule framed in purple in **C**, compare to **B**.

The contribution of the pair-based STDP rule includes the motif coefficient functions, $M_{\alpha ,\beta }^{pair}$, which are calculated in the Fourier domain (Eq 29 in Methods). The pairwise correlations between *i* and *j* also contribute to plasticity of *W*_*ij*_ based on the triplet STDP rule through the motif coefficient functions $M_{\alpha ,\beta }^{trip}$ (Eq 30 in Methods). Examples of some motifs common for both the pair-based and the triplet STDP rule are provided in Fig 3A. Their contribution to plasticity through the EPSC function *E* and the STDP rules *L*_2_ and *L*_3_ is illustrated in Fig 3B.

In addition to the *α* and *β* path lengths, to derive the contribution of the triplet STDP rule to the average change in synaptic efficacy, we also introduced the *γ*-path so that now motifs have order *α* + *β* + *γ*. *γ* is the synapse path length from the source neuron *k* to the postsynaptic neuron *i*, including a time delay relative to the *α* path from *k* to *i*, to account for the second postsynaptic spike of the triplet (Fig 3C and 3D). Thus, for the auto-covariance term in Eq 2, we obtain (Fig 2B)

$$\begin{array}{c} \left(**\right)\equiv \sum_{k=1}^{N}r_{k}\sum_{\alpha ,\gamma }{\left(W^{\alpha }\right)}_{ik}{\left(W^{\gamma }\right)}_{ik}r_{j}M_{\alpha ,\gamma }^{trip} \end{array}$$ (4)

where the motif coefficient function involving the triplet STDP rule is given in the Methods (Eq 31).

For third-order interactions, however, it is possible that the paths by which spikes are propagated branch out from a neuron other than the source neuron. Therefore, the third-order cumulant *K*_*ij*_ (Eq 2) is broken down into four sums:

$$\begin{array}{ll} \left(***\right)\equiv & \sum_{k=1}^{N}r_{k}\underset{\alpha ,\beta ,\gamma }{\sum }\overset{\text{No}\;\;\text{branching}\;\;\text{(straight}\;\;\text{paths)}}{\overset{︷}{{\left(W^{\alpha }\right)}_{ik}{\left(W^{\gamma }\right)}_{ik}{\left(W^{\beta }\right)}_{jk}M_{\alpha ,\beta ,\gamma }^{trip}}}\\ & \begin{array}{l} +\sum_{k,l=1}^{N}r_{k}\underset{\alpha ,\beta ,\gamma ,\zeta }{\sum }{\left(W^{\zeta }\right)}_{lk}{\left(W^{\alpha }\right)}_{ik}{\left(W^{\beta }\right)}_{jl}{\left(W^{\gamma }\right)}_{il}M_{\left(\alpha ,\zeta \right),\beta ,\gamma }^{trip}\\ +\sum_{k,l=1}^{N}r_{k}\underset{\alpha ,\beta ,\gamma ,\zeta }{\sum }{\left(W^{\zeta }\right)}_{lk}{\left(W^{\alpha }\right)}_{il}{\left(W^{\beta }\right)}_{jk}{\left(W^{\gamma }\right)}_{il}M_{\alpha ,\left(\beta ,\zeta \right),\gamma }^{trip}\\ +\sum_{k,l=1}^{N}r_{k}\underset{\alpha ,\beta ,\gamma ,\zeta }{\sum }{\left(W^{\zeta }\right)}_{lk}{\left(W^{\alpha }\right)}_{il}{\left(W^{\beta }\right)}_{jl}{\left(W^{\gamma }\right)}_{ik}M_{\alpha ,\beta ,(\gamma ,\zeta ).}^{trip} \end{array}\}\text{Branched}\;\text{paths} \end{array}$$ (5)

**Fig 4:**
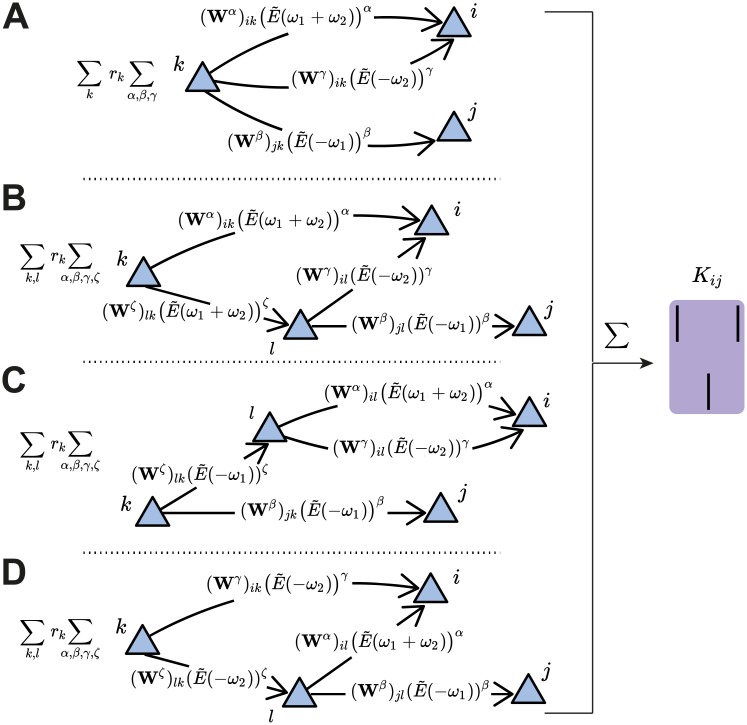
Third-order cumulant contributions to plasticity can be broken down into four terms. **A**. The first term contains all the *α*-, *β*- and *γ*-paths originating from the source neuron *k* to the spiking neurons *i* and *j*. **B-D**. The other terms take into account the possibility of an intermediate neuron *l* that acts as a new source neuron for two of the paths. These are referred to as ‘branched paths’, and the path length from the source neuron *k* to the intermediate neuron *l* is denoted with *ζ*. The branching describes the individual terms in Eq 5.

The first term in Eq 5 sums over the paths to the presynaptic neuron *j* and postsynaptic neuron *i* from a source neuron *k* in the network that do not branch out. In other words, it considers that the ‘distance’ to each respective spike of the triplet is given by *α*, *β* and *γ* (Fig 4A). The remaining terms include the sum over possible branches in the network ‘tree’: *ζ* ≥ 1 is the synapse path length from the source neuron *k* to the neuron *l* that is the branching point (Fig 4B–4D). It should be noted that the branched paths all have a total path length of at least four (i.e. *α* + *β* + *γ* + *ζ* ≥ 4) so that the motif order is at minimum four, since at least one synapse must be taken into account before the splitting of the path. The corresponding motif coefficients for the ‘straight’ triplet motif (Fig 4A, see Eq 32), and for the ‘branching’ motifs (Fig 4B–4D, see Eqs 33, 34 and 35) are provided in the Methods.

This analysis reveals novel motifs in the triplet STDP rule which have the potential to promote particular connectivity structures that are not possible with pair-based STDP [6] (Fig 3C). These include motifs which directly exclude the presynaptic neuron *j* but can still impact the synaptic weight, *W*_*ij*_ (Fig 3C, left and middle). This can be achieved, for example, through an additional neuron *k* that does not directly affect the weight *W*_*ij*_ but projects to the postsynaptic neuron *i* through the synaptic weight *W*_*ik*_ (Fig 3C, left and middle). Because these motifs exclude the presynaptic neuron *j*, they do not impact the pairwise cross-covariance term *C*_*ij*_ and do not have influence on the weight *W*_*ij*_ through pair-based STDP. For example, in the case when *α* = 2 and *γ* = 0 (Fig 3C, middle), the postsynaptic neuron *i* is both the source neuron and the neuron involved in the path with the additional neuron *k*. We call this path involving the synaptic efficacies *W*_*ik*_ and *W*_*ki*_ a ‘loop’. These loops involve a different neuron in addition to the pre- and postsynaptic neuron of the weight *W*_*ij*_, and are a unique feature of incorporating spike triplets in the STDP rule. Loops include a neuron as both the source and target for the spike in the corresponding path, so that a ‘loop’ closes on itself. The direction of the edges are relevant for this definition. We propose that motifs with these ‘loop’ characteristics promote the formation of connections between clusters of neurons, and therefore assemblies.

**Fig 5:**
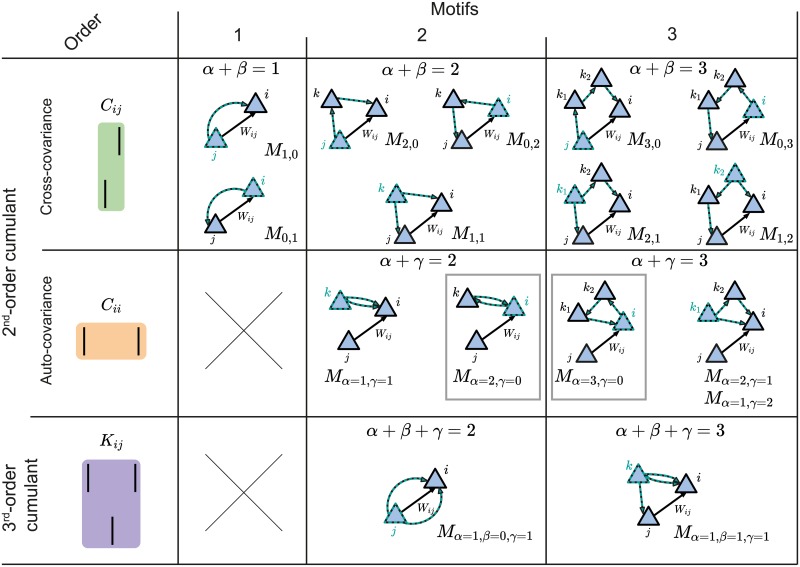
Second- and third-order cumulants can be described in terms of structural motifs that contribute to weight change. All motifs up to third order as they arise from the cross-covariance *C*_*ij*_ (top row and Eq 3), the auto-covariance *C*_*ii*_ (middle row and Eq 4) (both *C*_*ij*_ and *C*_*ii*_ together represent the second-order cumulant) or the third-order cumulant *K*_*ij*_ (bottom row and Eq 5). The gray boxes indicate the ‘loop’ motifs. The novel motifs which follow from the triplet STDP rule are those that include the path *γ* (second and third row).

To illustrate motifs of different orders and their relationship to cumulants of different orders, we depict all motifs up to third order arising from the expansion of the second- and the third-order cumulants (Fig 5). While it is clear that the full network connectivity through motifs of different orders from the cross-covariance *C*_*ij*_ influences plasticity under pair-based, as well as triplet STDP (Fig 5, first row), we also reveal novel motifs from the auto-covariance *C*_*ii*_ and the third-order cumulant *K*_*ij*_ that influence plasticity uniquely under triplet STDP (Fig 5, second and third row).

Taken together, our motif expansion framework reveals novel structural motifs under the triplet STDP rule that have the potential to form assemblies without structured external input. We next investigated the role of the different structural motifs (specifically the ‘loop’ motifs) on the emergence of assemblies under triplet STDP.

### Modulation of the triplet STDP rule promotes the autonomous emergence of assemblies

So far, we considered general STDP rules that depend on the precise timing between pairs and triplets of spikes, without taking into account the exact dependence of potentiation or depression on these spikes. To further study the complex relationship between plasticity and network correlations, we considered a particular biologically identified STDP rule that relies on third-order interactions (Methods; Fig 1C). This rule has an asymmetric shape around the time lag of 0 (where pre- and postsynaptic spikes are coincident), similar to the classical pair-based STDP rule [71]. However, while synaptic depression is induced by the relative timing of pairs of presynaptic and postsynaptic spikes, the minimal triplet STDP model uses sets of three spikes to induce potentiation: the amount depends on the timing between pre- and postsynaptic spike pairs and in addition, on the timing between the current and the previous postsynaptic spike (Fig 1D). This *minimal* model successfully captures experimental data, where the pairing frequency of pre- and postsynaptic spikes was varied, equally well compared to a full model that also uses triplets of spikes for depression [54].

**Fig 6:**
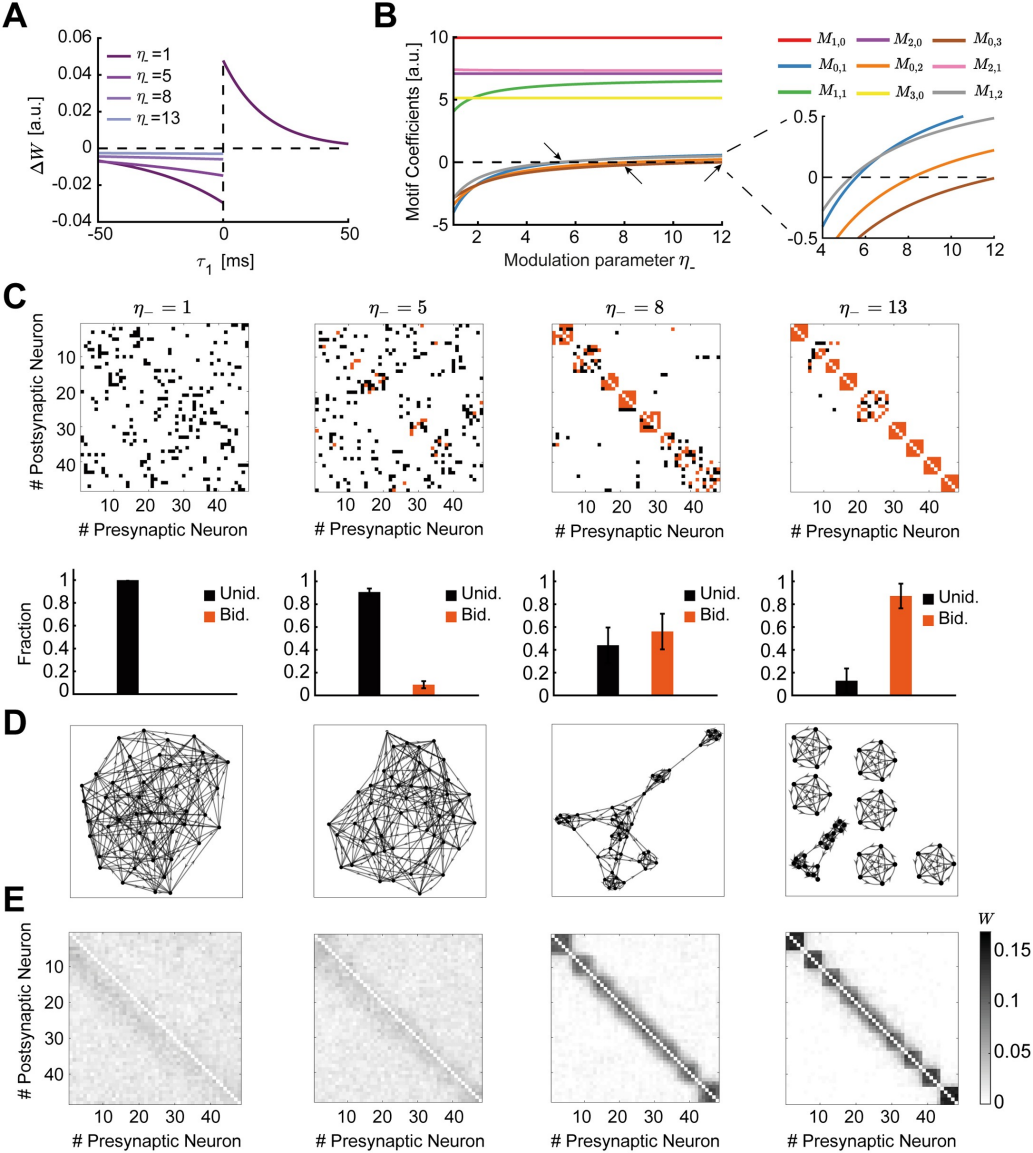
Spontaneous emergence of assemblies via modulation of the triplet STDP rule. **A**. The shape of the STDP function changes as a function of the modulation parameter *η*_−_, which preserves the overall level of depression by trading off the depression learning rate and the depression time constant. **B**. Motif coefficients as the modulation parameter *η*_−_ increases. Points of interest given by the crossovers of the strength of particular motifs are indicated by a small arrow. Inset: Amplified scale around zero. Motif coefficients including *γ* paths are not illustrated, since they are always constant and positive in *η*_−_ and do not provide a meaningful comparison to the other motifs. **C**. Top: Examples of connectivity matrices obtained with different values of *η*_−_ at steady state. Unidirectional connections are shown in black, bidirectional connections in orange. Matrices are reordered using the k-means clustering algorithm (see Methods). Bottom: Mean fraction of unidirectional and bidirectional connections for 100 trials with different initial synaptic efficacies as a function of *η*_−_. Error bars represent the standard error of the mean. **D**. Graphs of the connectivity matrices in C. **E**. Averaged connectivity matrices over 100 trials at steady state. Note that the tighter clusters emerging near the edges of the matrices are the result of the clustering algorithm but do not affect the quantification of connectivity.

Implementations of classical Hebbian learning, such as STDP, use joint pre- and postsynaptic activity to induce potentiation and depression, while neglecting other potential factors such as heterosynaptic plasticity [81], or the location of synaptic inputs on the dendritic tree [82]. However, recent experimental studies have highlighted an important role of neuromodulators in regulating plasticity across the brain [83–86], as they convey information about novelty or reward. Indeed, neuromodulators such as dopamine, acetylcholine and noradrenaline, but also brain-derived neurotrophic factor (BDNF) and gamma-aminobutyric acid (GABA), can predominantly act via two mechanisms: by reshaping the learning window for STDP or by regulating neuronal activity at the level of synaptic transmission [84, 86]. Therefore, we next investigated how neuromodulation of synaptic plasticity affects recurrently connected networks considering that pairwise and triplet spike interactions determine plasticity. We assume that the shape of the STDP function can be modulated via the modulation parameter *η*_−_ which preserves the overall level of depression by trading off the depression learning rate *A*_−_ and the depression time constant *τ*_−_ (Methods; Fig 6A). Such a modification of the learning rule has been observed in the lateral amygdala due to the action of dopamine via D2 receptors [85, 87], or in rat visual cortex slices with the activation of both the noradrenaline pathway through *β*-adrenergic receptors and the acetylcholine pathway through *M*_1_-muscarinic receptors [84, 86, 88]. A similar modulation parameter could similarly be included for potentiation.

To determine contributions to plasticity arising due to internal network correlations and not just differences in neuronal firing rates [5], we consider the case in which the plasticity rule is balanced, such that ${\tilde{L}}_{2}\left(0\right)+r_{i}{\tilde{L}}_{3}\left(0,0\right)=0$. We use this condition to calculate all motif coefficients, *M*_*α*,*β*_, that arise from the cross-covariance *C*_*ij*_ (Eqs 47–56 in Methods). We consider only motifs up to third-order in the evolution of the weights (Eq 2) since higher-than-third-order motif contributions are negligible (S1 Fig). Thus, we no longer include the branched path motifs of Eq 5 as they are higher-than-third-order motifs (Fig 4B–4D). This leaves us with a handful of motifs which arise from the second-order cumulant (consisting of the cross-covariance *C*_*ij*_ and the auto-covariance *C*_*ii*_) and the third-order cumulant *K*_*ij*_ (Methods; Fig 5). This simplification allows us to study the spontaneous emergence of assemblies under the triplet STDP rule based on both the triplet rule contributions to the cross-covariance *C*_*ij*_ (Eq 3, Fig 5, top row) and the influence of the novel branching structures that follow from the auto-covariance *C*_*ii*_ (Eq 4, Fig 5, second row, including the loop motifs in the gray boxes) and the third-order cumulant *K*_*ij*_ (Eq 5, Fig 5, third row).

To systematically study how the dependence of these up to third-order motif coefficients on the shape of the STDP rule affects connectivity structure in the network, we visualized the connectivity matrices obtained by integrating the motif expansion up to third-order (Eqs 42–46) numerically, using experimentally-fitted parameters for the triplet STDP rule and the EPSC function (Table 1). Specifically, we investigated the emergence of global network structures, or assemblies, as a function of the modulation parameter *η*_−_. This parameter has a direct influence on the motifs which follow from the cross-covariance *C*_*ij*_ (Fig 5) and the LTD window in the minimal triplet STDP rule (see Eqs 49–58 and S2 Text). A key requirement for the emergence of assemblies is the formation of bidirectional or reciprocal connections among groups of neurons. We compare the reciprocal connections of the first-order motif contributions to gain intuition:

$$\begin{array}{c} \begin{array}{cc} {\left\langle {\dot{W}}_{ij}^{\text{STDP}}\right\rangle }^{\left(1\right)} & =r_{j}W_{ij}M_{1,0}+r_{i}W_{ji}M_{0,1},\\ {\left\langle {\dot{W}}_{ji}^{\text{STDP}}\right\rangle }^{\left(1\right)} & =r_{i}W_{ji}M_{1,0}+r_{j}W_{ij}M_{0,1}. \end{array} \end{array}$$ (6)

Since in the triplet STDP rule *M*_1,0_ > 0 (Fig 6B, red), the two bidirectional connections compete if *M*_0,1_ < 0, and potentiate if *M*_0,1_ > 0. Therefore, the sign of the motif coefficient *M*_0,1_, which depends on the contribution from the triplet STDP rule, determines the formation of bidirectional connections. Indeed, increasing *η*_−_ supports the formation of bidirectional connections (Fig 6C) as the motif coefficient *M*_0,1_ changes sign (Fig 6B, blue, see inset). In contrast, as previously shown, the classical pair-based STDP rule is unable to support the formation of assemblies and bidirectional connections due to its asymmetric shape [89, 90], although under certain conditions (dominant potentiation) it can promote bidirectional connections [51, 91]. Under the asymmetric pair-based STDP rule, *M*_1,0_> and *M*_0,1_ < 0 result in competition between the two reciprocal connections. To autonomously generate self-connected assemblies without structured network input requires a symmetric pair-based STDP rule (which is not biologically motivated) and a sufficiently large synaptic latency [6]. In this case, the prominence of the common input motif driven by the *M*_1,1_ motif coefficient over all other motif coefficients in the network supports assembly formation [6].

**Table 1:** Parameter values for figures. ⋆ denotes that values are provided in the figures.

Symbol	Description	Fig 6	Fig 7	Fig 8	Fig 9	Fig 10	Fig 11
*N*	Number of neurons	48					
***μ***	External input firing rate	150 Hz					
*w*_*max*_	Upper bound for each individual weight	0.17					
*W*_*max*_	Upper bound for total row/ column weight	0.85					
*A*_−_	Depression learning rate	0.01					
*τ*_−_	Depression time constant	33.7 ms [54]					
*τ*_+_	Potentiation time constant	16.8 ms [54]					
*τ*_*y*_	Second potentiation time constant	114 ms [54]					
*η*_−_	Depression modulation parameter	⋆			1		⋆
*τ*_*ε*_	First membrane time constant	5 ms					
*τ*_*ι*_	Second membrane time constant	5 ms			⋆	5 ms	
*ν*	Scaling parameter of learning rate	3.5 ×10^−4^					
*ψ*	Heterosynaptic competition scaling parameter	0.7					

Under the triplet STDP rule, small increases in *η*_−_ increase the motif coefficient *M*_1,1_, resulting in the formation of bidirectional connections and assemblies, similarly to the symmetric pair-based STDP rule. However, despite its asymmetric shape, the triplet STDP rule can robustly generate bidirectional connections and assemblies even when the *M*_1,1_ motif coefficient has already saturated and other motif coefficients dominate (Fig 6C–6E), upon further increases in *η*_−_. This is because higher-order structural motifs also contribute to the formation of bidirectional connections and assemblies. To understand this, we consider the motif contributions of feedforward motif coefficients—the motifs for which the *α*-path is longer than the *β*-path, *M*_1,0_, *M*_2,1_, *M*_2,0_ and *M*_3,0_—and reciprocal motif coefficients, where the *β*-path is longer than the *α*-path, *M*_0,1_, *M*_1,2_, *M*_0,2_ and *M*_0,3_. Given the asymmetry of triplet STDP, the feedforward motif coefficients are stronger. The reciprocal motifs, *M*_0,1_, *M*_1,2_, *M*_0,2_ and *M*_0,3_ play an important role in the formation of bidirectional connections as they change sign from negative to positive with increasing *η*_−_ (Fig 6B). A positive contribution from all motifs supports the robust formation of bidirectional connections in the network as the competition between reciprocal connections decreases. Together with the strong common input motif driven by the *M*_1,1_ motif coefficient, this leads to the robust emergence of assemblies. In this scenario, *η*_−_ controls the competition between feedforward (*W*_*ji*_) and reciprocal connections (*W*_*ij*_), with large *η*_−_ enabling the potentiation of both. This is not possible under the classical asymmetric pair-based STDP rule as previously discussed.

In summary, we find that the spontaneous formation of self-connected assemblies depends on the modulation parameter *η*_−_, which influences most of the motifs arising from the cross-covariance *C*_*ij*_. Furthermore, self-connected assemblies can be formed under triplet STDP even when motifs other than the common input motif *M*_1,1_ dominate. This occurs despite the asymmetric shape of the triplet STDP rule, in contrast to pair-based STDP which requires a symmetric shape to promote *M*_1,1_. Importantly, the dependence of assembly formation on the specific form of the STDP window points towards an important role of neuromodulatory signals on formation of intrinsically generated assemblies.

### Characterizing emergent assembly structures

To determine the conditions on the learning rule and EPSC properties for the emergence of self-connected assemblies, it is convenient to represent the functional organization of the network given a connectivity matrix as a mathematical graph. In our context, graphs are composed of a set of nodes or neurons with pairs of them joined by edges or synaptic efficacies. The resulting graphs can be described by standard metrics, whose dependence on the shape of the learning rule and the EPSC function might yield insight into the emergent structures during circuit organization driven by spontaneous activity. We focused on common quantities for describing graph structure, including the clustering coefficient, the global efficiency and the modularity [92, 93], used previously in experimental systems like the zebrafish tectum and the mammalian cortex [25, 94].

**Fig 7:**
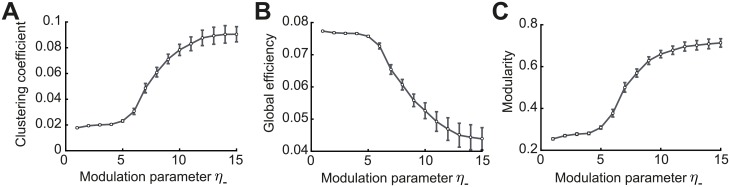
Graph measures of the stable network configuration. **A**. Mean clustering coefficient versus the modulation parameter *η*_−_. **B**. Mean global efficiency versus the modulation parameter *η*_−_. **C**. Mean modularity versus the modulation parameter *η*_−_. All results are calculated from 100 trials at steady state connectivity. Error bars represent the standard error of the mean.

The clustering coefficient quantifies the existence of densely interconnected groups of nodes in the graph [95]. It represents a measure of segregation, based on counting the number of connection triangles around a node (Methods). In this manner, it reflects the prevalence of clustered connectivity around individual nodes by calculating the fraction of neighbors of that particular node that are also neighbors of each other. As a result, the mean clustering coefficient of a network determines the prevalence of three-neuron-clusters in the network architecture. We find that as the modulation parameter *η*_−_ increases, the mean clustering coefficient also increases until it reaches a plateau (Fig 7A). Ensuring that the motif coefficients *M*_0,1_ and *M*_1,2_ are positive is sufficient for the formation of clusters beyond the critical value of *η*_−_ ≈ 5 (Fig 6B and 6C), where the clustering coefficient begins to increase (Fig 7A). The value of *η*_−_ at which the clustering coefficient saturates corresponds to the emergence of more robust assemblies where all the motif coefficients are positive (Fig 6B and 6C). Although strong bidirectional connections are localized within clusters, connections from one cluster to some others still exist globally. This is different to the clustering enabled by strong symmetric interactions in which the motif *M*_1,1_ dominates, considered previously by a symmetric pair-based STDP rule [6], where the clusters would be unconnected (i.e. isolated from each other) and the clustering coefficient would be much higher.

Complementary to the clustering coefficient, the global efficiency is a measure of functional integration, which determines how easily nodes can communicate between each other through sequences of edges [96]. Consequently, the lengths of the paths estimate the potential for the flow of information between nodes, with shorter paths denoting stronger capacity for integration. Then, global efficiency is defined as the average inverse shortest path length of the network (Methods). In comparison to the clustering coefficient, this quantity initially remains approximately constant and then decreases until the point at which strong assemblies emerge autonomously since network structure no longer varies with the parameter *η*_−_ (Fig 7B). We find that as for the clustering coefficient, the value of *η*_−_ for which the motif coefficients *M*_0,1_ and *M*_1,2_ become positive (*η*_−_ ≈ 5) constitutes a landmark for the formation of assemblies, after which global efficiency significantly decreases.

Finally, modularity is a graph theoretic measure that describes how strongly a network can be divided into modules, by comparing the relative strengths of connections within and outside modules to the case when the network has randomly chosen weights [93, 97, 98]. Recently, it was shown that even in models with rate-based dynamics, increasing modularity amplifies the recurrent excitation within assemblies evoking spontaneous activation [48]. With increasing *η*_−_, modularity increases until strong assemblies are formed in a similar fashion as the clustering coefficient (Fig 7C). Interestingly, the critical value of *η*_−_ ≈ 5 where assemblies begin to form robustly is consistent with experimental evidence of the shape of STDP where the time constant for depression has been found to be approximately 5 times longer than for potentiation [55, 99].

### Contribution of the novel structural motifs under triplet STDP on assembly formation

So far we demonstrated that the spontaneous emergence of assemblies via modulation of triplet STDP depends on the interaction of different motifs that primarily arise from the second-order cross-covariance *C*_*ij*_ (Figs 5 and 6), which is also present under pair-based STDP. However, whether the novel structural motifs that are unique to triplet STDP (Figs 2B and 3–5) play a role remains unclear. We hypothesize that the ‘loop’ motifs, which do not appear for the pair-based STDP rule (Fig 5; gray box) are important for assembly formation.

**Fig 8:**
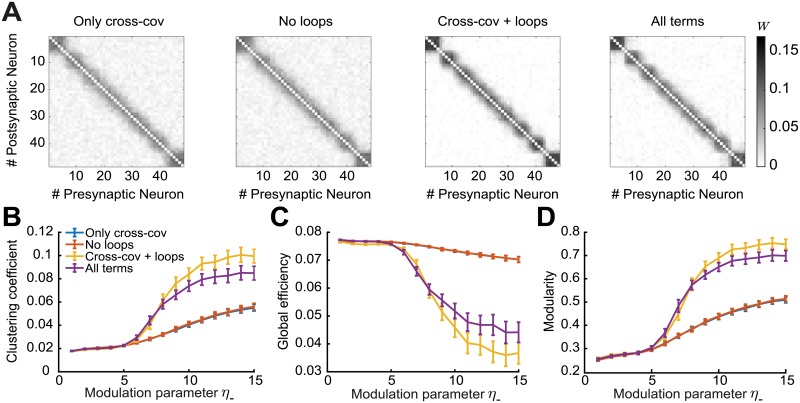
Spontaneous emergence of assemblies for four different motif combinations. Considering only motifs related to the cross-covariance *C*_*ij*_ (blue), from all cumulants (*C*_*ij*_, *C*_*ii*_ and *K*_*ij*_) without the ‘loop’ terms (red), from the cross-covariance *C*_*ij*_ plus the ‘loop’ terms (yellow) and from all cumulants (purple). **A**. Averaged connectivity matrices over 100 trials at steady state for four different motif combinations and modulation parameter *η*_−_ = 13. Matrices are reordered using the k-means clustering algorithm (see Methods). **B**. Mean clustering coefficient versus the modulation parameter *η*_−_. **C**. Mean global efficiency versus the modulation parameter *η*_−_. **D**. Mean modularity versus the modulation parameter *η*_−_. All results are calculated from 100 trials at steady state connectivity. Error bars represent the standard error of the mean.

To investigate the implications of these novel ‘loop’ motifs, we compare the three graph measures in four different scenarios: Using the motifs (1) only from the cross-covariance *C*_*ij*_ (Fig 5, top row); (2) from all cumulants (*C*_*ij*_, *C*_*ii*_ and *K*_*ij*_) without the ‘loop’ terms (Fig 5 all except the gray boxes in the second row); (3) from the cross-covariance plus the two additional ‘loop’ terms (Fig 5, top row plus the gray boxes in the second row); and (4) from all cumulants (Fig 5, all). We find that cases (1) and (2) have worse performance in all three graph measures compared to cases (3) and (4) (Fig 8). Adding the third-order cumulant *K*_*ij*_ and the ‘non-loop’ terms from the second-order auto-covariance *C*_*ii*_ (case 4) even worsens the graph measures. We find that the third-order cumulant *K*_*ij*_ alone has almost no influence on the spontaneous emergence of assemblies (S2 Fig), since its contribution to the weight change is small, as shown before [100]. We conclude that the additional ‘loop’ terms, which arise as novel structural motifs from the triplet STDP rule (Fig 5), have a significant contribution to spontaneous assembly formation.

### The triplet STDP rule and the EPSC together modulate network structure

**Fig 9:**
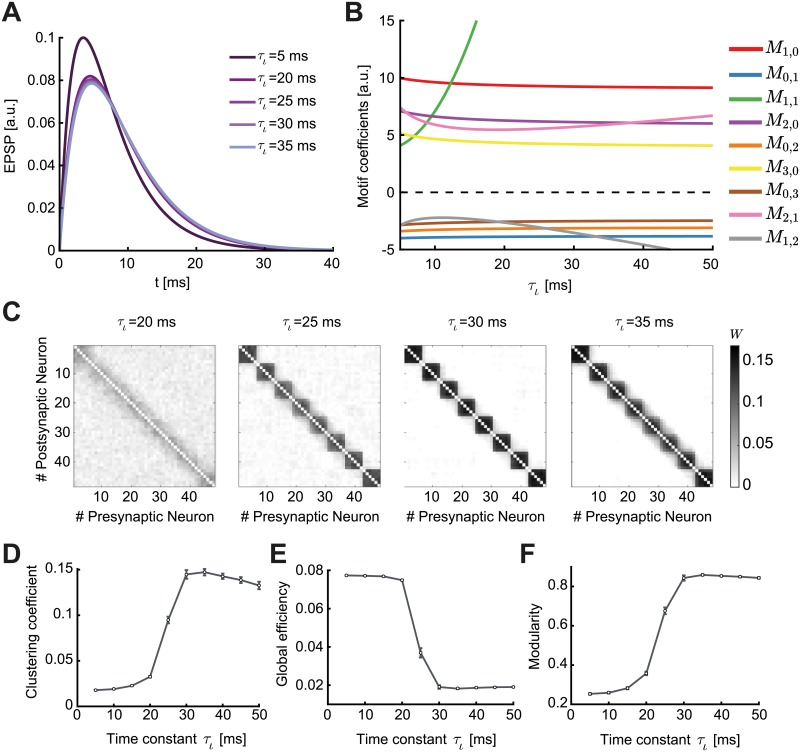
Spontaneous emergence of assemblies due to the modulation of synaptic transmission. **A**. Varying the time constant *τ*_*ι*_ changes the shape of the EPSC function, shifting its peak by a few milliseconds. **B**. Relative value of the motif coefficients as a function of *τ*_*ι*_. While the common input motif *M*_1,1_ rapidly assumes dominance, the motif coefficient *M*_1,2_ crosses over in strength with the feedback motifs *M*_0,1_, *M*_0,2_ and *M*_0,3_. **C**. Averaged connectivity matrices over 100 trials at steady state and different values of the time constant *τ*_*ι*_. Matrices are reordered using the k-means clustering algorithm (see Methods). **D**. Mean clustering coefficient versus the time constant *τ*_*ι*_. **E**. Mean global efficiency versus the time constant *τ*_*ι*_. **F**. Mean modularity versus the the time constant *τ*_*ι*_. All results are calculated from 100 trials at steady state connectivity. Error bars represent the standard error of the mean.

The spontaneous emergence of assemblies discussed so far requires a relatively high value of the STDP modulation parameter *η*_−_, raising the issue of biological plausibility. Although several experimental studies on induction of STDP indeed find longer depression than potentiation time constants [55, 99], we demonstrate an alternative mechanisms for the assembly formation by regulating the synaptic transmission of action potentials between neurons through the shape of the EPSC function. In this case, the strength of internally generated correlations can be changed independently of the STDP functions, *L*_2_ and *L*_3_. We investigated how the rise of the EPSC function modulated by delay of the spike transmission in the synapse, *τ*_*ι*_ (Fig 9A), shapes motif coefficients (Methods; Fig 9B).

The parameter *τ*_*ι*_ has a prominent impact on plasticity in the network. Even small shifts in the peak of the EPSC function by a few milliseconds have a strong impact on the cumulants of different orders, as reflected in the values of the motif coefficients (Fig 9A and 9B). Different to the modulation with the parameter *η*_−_, the parameter *τ*_*ι*_ affects all motif coefficients. However, the influence of *τ*_*ι*_ on the auto-covariance *C*_*ii*_ and the third-order cumulant *K*_*ij*_ is negligible. Therefore, although the main cumulant driving plasticity is the second-order cross-covariance *C*_*ij*_, which exists even under pair-based STDP (Fig 5), assemblies easily form under the triplet STDP rule (Fig 9C). The common input motif *M*_1,1_ abruptly assumes dominance over all others as *τ*_*ι*_ increases (Fig 9B). However, we observed that the reciprocal motif coefficients *M*_0,1_, *M*_1,2_, *M*_0,2_ and *M*_0,3_ remain negative for all values of *τ*_*ι*_, in contrast to when we modulated the STDP learning rule (Fig 6B). This tells us that assemblies in the network are spontaneously formed in a different fashion (by promoting the potentiation of reciprocal connections in each cluster due to the common input motif, *M*_1,1_) than when modulating the STDP rule through *η*_−_. In fact, assemblies emerge for minor modulations in *τ*_*ι*_ (Fig 9C).

These differences in assembly formation become apparent when we consider the mean clustering coefficient, the global efficiency and the modularity as functions of *τ*_*ι*_ (Fig 9D–9F): the three measures reflect the connectivity matrices as *M*_1,2_ crosses the motif coefficients *M*_0,1_, *M*_0,2_ and *M*_0,3_, in the case when *M*_1,1_ is already large. When the motif coefficient *M*_1,2_ becomes more negative than *M*_0,3_ (*τ*_*ι*_ ≈ 20 ms), bidirectional connections are strongly promoted and assemblies robustly form. Even for *τ*_*ι*_ > 20 ms, where the EPSC function does not change significantly (Fig 9A), one sees noticeable changes in the ‘tightness’ of the assemblies as observed in the averaged connectivity matrices (Fig 9C). Interestingly, as *M*_1,2_ decreases below *M*_0,2_ (*τ*_*ι*_ ≈ 25 ms), the value of the clustering coefficient (≈ 0.1) and the modularity (≈ 0.7) correspond to the values where the clustering coefficient, the modularity, and the global efficiency saturate when modulating the STDP function (compare Figs 7 and 9D–9F). This means that the network structure is very similar (compare Fig 6E, right, with Fig 9C, second from left). Nevertheless, further increasing *τ*_*ι*_ leads to more refined assemblies (Fig 9C, third from left) when *M*_1,2_ < *M*_0,2_. However, for *τ_ι_* ≳ 35 ms where *M*_1,2_ < *M*_0,1_, the clustering coefficient slightly decreases (Fig 9D) suggesting the existence of optimal regions in the parameter space of *τ*_*ι*_ to obtain the ‘tightest’ assemblies.

Taken together, our analytical framework enables us to interpret changes in the motif coefficients as changes in the connectivity structure in terms of the formation of self-organized assemblies. Modifying either the shape of the learning rule, or the shape of the EPSC function, can achieve this, however, with different consequences on the nature of the formed structures as demonstrated by the graph theoretic measures.

### Comparison with assemblies generated via external correlated input

Until now, we sought to understand the mechanisms that contribute to the autonomous emergence of assemblies in neural circuits without any structured external input. Yet, the training of assemblies and plasticity of recurrent connections has been more frequently studied when these networks are driven by structured external input, both in simulations [49, 89] and analytically [42–45, 51]. Significant experimental evidence also exists for the emergence of functional connectivity underlying feature selectivity in the visual cortex around the time of eye opening, which is presumably influenced by structured visual input through the open eyes [14]. Therefore, we wanted to compare the formation of assemblies without structured external input under the triplet STDP rule to that with structured external input. To investigate spatiotemporal input patterns in our framework, we studied the overall mean impact of an external pairwise correlated input. This was implemented by assuming that the driving signal, which could for instance represent retinal input in the optic tectum or visual cortex, is correlated for a pair of neurons in the network, so that the structure of the input is represented as common input to that particular pair of neurons.

We write the covariance as a sum of the internal correlation and a novel term that conveys the external structured activity as common input [40]:

$$\begin{array}{c} \tilde{C}\left(\omega \right)={\tilde{C}}^{\text{int}}\left(\omega \right)+{\left(I-\tilde{E}(\omega )W\right)}^{-1}\tilde{E}\left(\omega \right){\tilde{C}}^{\text{ext}}\left(\omega \right)\tilde{E}\left(-\omega \right){\left(I-\tilde{E}\left(-\omega \right)W^{T}\right)}^{-1}. \end{array}$$ (7)

Here, ***C***^int^ denotes the covariance matrix (see Eq 22) and ***C***^ext^ is the covariance matrix of the external input. We model the input signal as a correlated pattern that promotes the joint activity of pairs of neurons that belong to a certain assembly.

**Fig 10:**
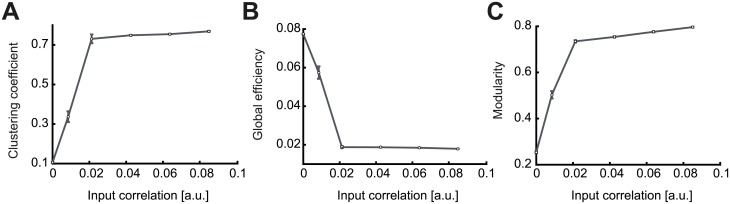
Emergence of assemblies in the presence of structured external input. **A**. Mean clustering coefficient versus the pairwise correlation coefficient of the input pattern. The strength of the correlation was provided as ratios (0.01, 0.05, 0.125, 0.25, 0.375 and 0.5) of the possible maximum weight of each individual synaptic connection *w*_*max*_. **B**. Mean global efficiency versus the pairwise correlation coefficient of the input pattern. **C**. Mean modularity versus the pairwise correlation coefficient of the input pattern. The rapid increase of the clustering coefficient and the modularity combined with a decrease of the global efficiency is a feature of robust assembly formation. Sufficiently strong correlations in the external signal generate tight assemblies. All results are calculated from 100 trials at steady state connectivity. Error bars represent the standard error of the mean.

Using the standard parameters of the minimal triplet model (Table 1; Fig 9C, *η*_−_ = 1) assembly formation is difficult when the feedforward motif coefficients dominate (the motifs for which the *α*-path is longer than the *β*-path). However, a significantly stronger external correlation relative to internally generated network correlations can promote the common input motif, *M*_1,1_, and support assembly formation. As a function of the external correlation matrix, we quantify the structure of the resulting self-connected assemblies of neurons via the same graph measures used previously (Fig 10). The tight assemblies observed for the modulation of the STDP and the EPSC functions (Figs 6E and 9C) can now be formed for values of correlation strength one order of magnitude smaller than the synaptic upper bound.

### Disrupting the balance between potentiation and depression affects assembly formation

We considered an STDP rule that is balanced in the total potentiation and depression, because disrupting this balance by increasing some firing rater over others favors the particular circuit motifs affected by those rates, as shown before [5, 45, 101]. When the balance is disrupted, the firing rate contribution to plasticity from chance spike coincidences dominates over internal correlations. When the zero-order term of the motif expansion (Eq 42) is non-zero, the mean change in the synaptic efficacies has a term that only depends on the firing rates. In this case, the firing rates of the pre- and postsynaptic neurons are the main drivers of network structure. This means that the overall impact of motifs in the network is diminished [5]. We explored the possible departures from balance through the inclusion of a perturbation parameter *δ* that can be either positive or negative and we scaled this parameter in proportion to the learning rate (Methods).

**Fig 11:**
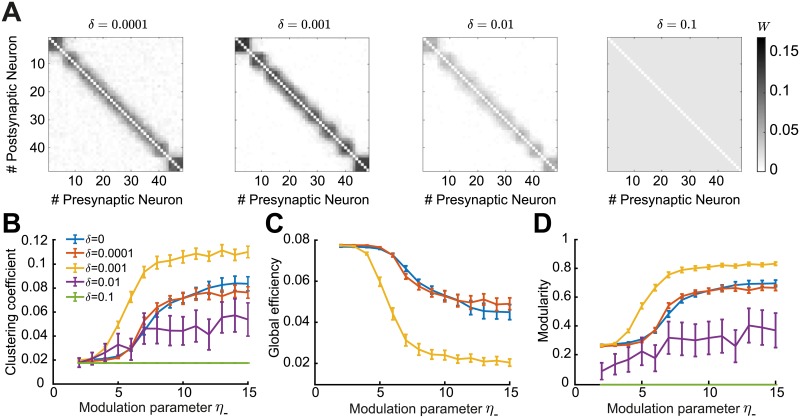
Impact of perturbations in the balance of potentiation and depression of the triplet STDP rule. **A**. Averaged connectivity matrices over 100 trials at steady state for the four different cases of the perturbation parameter *δ* and modulation parameter *η*_−_ = 13. **B**. Mean clustering coefficient versus the modulation parameter *η*_−_. **C**. Mean global efficiency versus the modulation parameter *η*_−_. We removed the cases *δ* = [0.01, 0.1] here since the global efficiency cannot be computed for weight matrices where all entries are identical. **D**. Mean modularity versus the modulation parameter *η*_−_. All results are calculated from 100 trials at steady state connectivity. Error bars represent the standard error of the mean.

Therefore, to study the sensitivity of the emergence of network structure to perturbations on the depression vs. potentiation balance we consider that the zero-order ‘rate’ motif is different from zero. We find that departures from the balanced regime impact plasticity significantly. In the case of a depression dominated imbalance, *δ* < 0, all connections depress no matter the strength of the modulation through *η*_−_, even for small absolute value of *δ* = −0.0001. In the case of potentiation, *δ* > 0, one might expect that all synaptic efficacies will just saturate; however, due to heterosynaptic competition, some network structure still forms when *δ* is small (Fig 11). If the perturbation is sufficiently strong, the autonomous emergence of assemblies by increasing the parameter *η*_−_ is disrupted. This is also evidenced when computing the graph measures for the resulting network structures (Fig 11B–11D). In summary, we find that considering an unbalanced STDP rule where either depression or potentiation dominates, prevents the autonomous emergence of assemblies.

## Discussion

We developed a self-consistent theoretical framework to study the impact of HOCs, specifically up to third order, on the plasticity of recurrent networks by using the triplet STDP rule. We derived the dependence of the drift in synaptic efficacy on network structure, taking into account contributions from structural motifs of different orders, and demonstrated the emergence of global network structures i.e. assemblies, from these local motifs. Based on recent work on the calculation of beyond second-order cumulants of mutually exciting Hawkes processes [37, 102], we broke down the spike interactions (including pairs and triplets of spikes) to include the influence of spikes from any source neuron in the network on the firing of the pre- and postsynaptic neurons via paths of different length thus taking into account the full network recurrence (Figs 2 and 4). We characterized structural motifs that arise from these spike interactions, including novel motifs arising due to triplet STDP, and analyzed their impact on the internal up to third-order correlation structure and plasticity in the network through the motif coefficients (Figs 3 and 5). While linearization of neuronal dynamics was required for this approach, it is a common technique used to approximate the dynamics of more realistic biophysical neurons [5, 34]. We found that motif contributions to plasticity from the second-order cross-covariance *C*_*ij*_ support assembly formation under triplet STDP. However, since these same motifs exist also under pair-based STDP, we wondered if the novel motifs unique to triplet STDP are important for assembly formation. Indeed, we showed that several novel motifs and specifically the ‘loop’ motifs, which emerge under the triplet STDP, have an important contribution to the formation of assemblies (Fig 8).

We investigated the contribution of up to third-order structural motifs on assembly formation using an asymmetric minimal triplet STDP rule, in which depression is induced by pairs of spikes and, conversely, potentiation is induced by triplets of spikes (Fig 1D). This rule has been shown to describe plasticity experiments that the classical STDP rule, based on pairs of spikes, has failed to capture; for instance, plasticity experiments in which the pairing frequency during plasticity induction was varied [54, 55]. As such, the triplet STDP rule is sensitive to third-order correlations, here referred to as HOCs. HOCs have not only been measured in the brain, but also shown to play an important role in visual coding and representing experimental data [58, 59, 103, 104]. HOCs are ubiquitous in sensory stimuli, such as natural stimuli and speech signals [105, 106]. These correlations have been previously utilized in learning rules, such as the BCM rule, to extract the independent components or features in natural images resulting in simple cell receptive fields as seen in V1 [105, 107–109]. Because of its mapping to the BCM rule [57], we can interpret the triplet STDP rule as a method for performing similar computations.

Modulating either the STDP rule (Fig 6) or the EPSC function (Fig 9) enabled the spontaneous formation of self-connected assemblies without the need for externally patterned inputs [49–51] or assuming a symmetric pair-based STDP rule [6]. We quantified the nature of the emergent assemblies using three graph theoretic measures used to characterize spontaneous assemblies in the tectum of zebrafish larvae [25]. Directly comparing the values of these measures between the experimental data and our model results is difficult given inhomogeneities in the size of biological network assemblies and a multitude of mechanisms that shape their formation during development. Yet, comparing how these measures change as a function of the STDP rule or the EPSC kernel in our model could offer insights into how modulating plasticity and synaptic transmission affect network structure through spontaneous activity under minimal assumptions (Fig 7). Interestingly, the final assemblies formed by modulating the EPSC function were more consistent across networks with different initial connectivity than the assemblies generated through the modification of the STDP function. This could be seen by the ‘tighter’ structures in the average connectivity matrices (Figs 6E and 9C), and the higher values of graph measures (Figs 6 and 9D–9F). The ultimate connectivity structure was determined by the relative strength of motifs which were regulated differently by each modulatory mechanism. In particular, modifying the EPSC function reinforced the influence of the common input motif (driven by the motif coefficient *M*_1,1_) over all others (Fig 9B). In comparison, the modulation of the STDP rule by extending the time constant for depression over potentiation reduced the competition between reciprocal connections by maintaining a strong feedforward drive (driven by the feedforward motif coefficients *M*_1,0_, *M*_2,1_, *M*_2,0_ and *M*_3,0_) and making the corresponding reciprocal motif coefficients (*M*_0,1_, *M*_1,2_, *M*_0,2_ and *M*_0,3_) positive. Therefore, assembly formation was driven by the strengthening of reciprocal connections, even though the *M*_1,1_ coefficient was still strong (Fig 6B). Although experimental evidence exists for a longer time constant for depression over potentiation in STDP [55, 99], the much longer values of the STDP modulation parameter *η*_−_ needed for our results raise the question of whether this mechanism is biologically plausible. This might make the modulation of the EPSC function under triplet STDP more suitable for explaining the autonomous emergence of self-connected assemblies. It is probably the case that both mechanisms are used in biological circuits. Studying the effects of neuromodulation, which can alter the shape of STDP or the synaptic transmission function, on the plasticity of connections in many brain regions is possible with recent advances in experimental techniques [84–86]. Understanding the consequences of changing the properties of the underlying plasticity mechanisms on network dynamics can further elucidate the process of learning and memory storage in recurrent networks found everywhere in the brain [84–86].

Applying external correlated input led to the emergence of self-organized assemblies (Fig 10) that were similar to the assemblies from changing the EPSC function. Consequently, we propose that the mechanisms that promote the formation of assemblies can be diverse in different circuits depending on the nature of the plasticity rules, synaptic transmission (EPSC function) or the structure of external input that dominate in these circuits.

Our framework enabled us to derive global connectivity structures that emerge in recurrent networks such as assemblies, which have been abundantly observed in experimental data. Connectivity matrices of large recurrent networks are generally difficult to assay experimentally, requiring multiple cells to be patched simultaneously [110], although recent developments in the field of connectomics offer potential for these matrices to be obtained in the future [111, 112]. However, a good experimental determinate of assemblies may be derived from functional interactions among neurons, inferred from physiological experiments that simultaneously record the activity of a large number of neurons. While it is clear that neuronal activity exhibits structure in response to sensory input, assemblies are present even during spontaneous activity and have similar spatial organization [21, 25, 26]. This has suggested that these self-organized assemblies are biologically relevant for the processing of information in these networks and for the representation of sensory stimulus attributes [21]. In the rodent visual cortex, a given stimulus, of the form of a natural scene or an orientated grating, consistently activates a specific assembly [21]. On the behavioral scale, recent experiments suggest that functional circuit connectivity may be intrinsically adapted to respond preferentially to stimuli of biological relevance for the survival of the animal, such as catching prey or avoiding predators [24, 27].

Our analytical approach offers a precise description of how synaptic plasticity shapes connectivity in recurrent networks driven by spontaneous activity (though we also considered the role of structured external input). Such spontaneous activity is especially common during early postnatal development, where it activates neural networks before the onset of sensory experience and the maturation of sensory organs. In the rodent visual system, for instance, eye opening only occurs during the second postnatal week of development [113]. Prior to this, spontaneous patterns of activity propagate throughout the entire visual system, including the retina, thalamus and cortex [114], which are known to instruct different aspects of circuit organization [115]. Interestingly, during very early postnatal development of somatosensory cortex in rodents (postnatal day 4), spontaneous activity exhibits a highly correlated state consisting of cell assemblies where multiple neurons show correlated activity [116]. By the second postnatal week this spontaneous activity transitions to a much more decorrelated state that lacks a clear spatial structure. A similar sparsification of spontaneous activity during development is also observed in the visual cortex, though lacking the spatial structure observed in the somatosensory cortex [117]. Since these two studies argue that over development functional connectivity becomes more desynchronized, this framework is more consistent with our analysis of the depression window of the STDP rule becoming smaller over development (Fig 6). This broadening of the depression window in early development is consistent with a previously described burst-timing-dependent plasticity where the temporal integration of activity occurs over much longer timescales on the order of several hundred milliseconds than in adulthood [115, 118, 119].

Assembly formation has been the goal of many other previous works, typically instructed by externally structured input in recurrent network models with balanced excitation and inhibition [42–47]. These assemblies exhibit attractor dynamics which have been argued to serve as the substrate of different computations, such as predictive coding through the spontaneous retrieval of evoked response patterns [49, 50, 120]. We investigated the generation of assemblies through triplet STDP driven by higher-order correlations generated internally in the network. Other works have also studied the emergence of non-random structure in the absence of structured external input [5, 6, 48]; our work takes a similar approach of incorporating the full recurrence in the network through the expansion into structural motifs as [6]. As it becomes evident from these studies, the investigation of STDP in recurrent networks for unsupervised learning involves a lot of parameters and additional mechanisms (including short-term plasticity, heterosynaptic plasticity and inhibitory plasticity) which make the identification of general rules difficult. Nevertheless, the precise theoretical description of triplet STDP in recurrent networks provided by our framework highlights a set of novel motifs absent in the case of pair-based STDP that promote assembly formation, in the process highlighting an important role of HOCs in the generation of global network structure from local motifs.

## Methods

### Network dynamics

The time dependent activity of a neuron *i* is given by a stochastic realization of an inhomogeneous Poisson process [70], with expectation value

$$\begin{array}{c} \lambda_{i}\left(t\right)=\mu_{i}+\sum_{k=1}^{N}W_{ik}\left[E*S_{k}\right]\left(t\right), \end{array}$$ (8)

where *μ*_*i*_ is the external input firing rate, ***W*** is the synaptic weight matrix, *S*(*t*) is the spike train and *E*(*t*) is the EPSC function, which we assume to be identical for all *N* neurons. Then, the product ***W**E*(*t*) is referred to as the interaction kernel. The operator ‘*’ corresponds to the convolution operation. In all plasticity simulations, the connectivity weight matrix is divided into an excitatory and an inhibitory component, such that the effective connectivity matrix is calculated as ***W***^eff^ = ***W*** − ***W***^inh^. The inhibitory weight matrix ***W***^inh^ is updated to balance the excitatory (see section ‘Additional plasticity mechanisms besides STDP’). For simplicity in notation, we refer to ***W***^eff^ as ***W*** in the manuscript.

### Averaged synaptic efficacy dynamics for pair-based and triplet STDP rules

Plasticity of the connectivity matrix ***W*** is determined by pair-based and triplet STDP rules. We assume ‘all-to-all’ interactions between spikes, where each postsynaptic spike interacts with every previous pre- and postsynaptic spike and vice-versa [52, 121–123].

Plasticity due to the pair-based STDP can be expressed as:

$$\begin{array}{c} \begin{array}{cc} {\dot{W}}_{ij}^{\text{pair}\;\text{STDP}}\left(t\right) & =\int_{-\infty }^{\infty }S_{i}\left(t\right)S_{j}\left(t-\tau_{1}\right)L_{2}\left(\tau_{1}\right)\text{d}\tau_{1} \end{array} \end{array}$$ (9)

and plasticity due to the triplet STDP rule as:

$$\begin{array}{c} \begin{array}{cc} {\dot{W}}_{ij}^{\text{triplet}\;\text{STDP}}\left(t\right) & =\overset{\infty }{\underset{-\infty }{\;\int \int \;}}S_{i}\left(t\right)S_{j}\left(t-\tau_{1}\right)S_{i}\left(t-\tau_{2}\right)L_{3,y}\left(\tau_{1},\tau_{2}\right)\text{d}\tau_{1}\;\text{d}\tau_{2}\\ & +\overset{\infty }{\underset{-\infty }{\;\int \int \;}}S_{j}\left(t\right)S_{i}\left(t-\tau_{1}\right)S_{j}\left(t-\tau_{3}\right)L_{3,x}\left(-\tau_{1},-\tau_{3}\right)\text{d}\tau_{1}\;\text{d}\tau_{3} \end{array} \end{array}$$ (10)

*L*_2_ corresponds to the pair-based STDP rule and *L*_3_ to the triplet STDP rule. The additional subscripts *x* and *y* denote that the triplets which contribute to plasticity are two pre- and one postsynaptic spikes and one pre- and two postsynaptic spikes, respectively. *τ*_1_ is the time difference between the spikes of the pre- and the postsynaptic neuron. *τ*_2_ is the time difference between two postsynaptic spikes and *τ*_3_ is the time difference between two presynaptic spikes (Fig 1B). It should be highlighted that this derivation is independent of the specific shape of the STDP functions.

Assuming slow learning in comparison to neuronal dynamics and that pairs and triplets of spikes between the pre- and postsynaptic neurons are relevant to plasticity [54, 57], the mean evolution of the synaptic efficacies due to STDP is given by

$$\begin{array}{c} \begin{array}{cc} \left\langle {\dot{W}}_{ij}^{\text{STDP}}\left(t\right)\right\rangle & =\int_{-\infty }^{\infty }\left\langle S_{i}\left(t\right)S_{j}\left(t-\tau_{1}\right)\right\rangle L_{2}\left(\tau_{1}\right)\text{d}\tau_{1}+\overset{\infty }{\underset{-\infty }{\;\int \int \;}}\left\langle S_{i}\left(t\right)S_{j}\left(t-\tau_{1}\right)S_{i}\left(t-\tau_{2}\right)\right\rangle L_{3,y}\left(\tau_{1},\tau_{2}\right)\text{d}\tau_{1}\;\text{d}\tau_{2}\\ & +\overset{\infty }{\underset{-\infty }{\;\int \int \;}}\left\langle S_{j}\left(t\right)S_{i}\left(t-\tau_{1}\right)S_{j}\left(t-\tau_{3}\right)\right\rangle L_{3,x}\left(-\tau_{1},-\tau_{3}\right)\text{d}\tau_{1}\;\text{d}\tau_{3} \end{array} \end{array}$$ (11)

where 〈⋅〉 denotes averaging over different realizations of the Poisson neuronal dynamics for different connectivity.

We define the mean rates of the pre- (*j*) and postsynaptic neuron (*i*) as *r*_*j*_ and *r*_*i*_. We consider both to be stationary at equilibrium. The second-order correlation between the pre- and postsynaptic neurons with time delay *τ*_1_ is 〈*S*_*i*_(*t*)*S*_*j*_(*t* − *τ*_1_)〉 and we define the covariance matrix (second-order cumulant) *C* (Fig 1B) as

$$\begin{array}{c} C_{ij}\left(\tau_{1}\right)=\left\langle S_{i}\left(t\right)S_{j}\left(t-\tau_{1}\right)\right\rangle -r_{i}r_{j}. \end{array}$$ (12)

Note that [6, 37] use a different convention for signs.

The third-order correlation between the triplet of spikes ‘post-pre-post’ with time delays between the pre and one post *τ*_1_ and between the two post *τ*_2_ is 〈*S*_*i*_(*t*)*S*_*j*_(*t* − *τ*_1_)*S*_*i*_(*t* − *τ*_2_)〉 and we define the third-order cumulant as [57]

$$\begin{array}{c} K_{ij}\left(\tau_{1},\tau_{2}\right)=\left\langle S_{i}\left(t\right)S_{j}\left(t-\tau_{1}\right)S_{i}\left(t-\tau_{2}\right)\right\rangle -r_{i}(C_{ij}\left(\tau_{1}\right)+C_{ij}\left(\tau_{2}-\tau_{1}\right))-r_{j}C_{ii}\left(\tau_{2}\right)-r_{i}^{2}r_{j}. \end{array}$$ (13)

Analogously, for the ‘pre-post-pre’ third-order correlation 〈*S*_*j*_(*t*)*S*_*i*_(*t* − *τ*_1_)*S*_*j*_(*t* − *τ*_3_)〉, we can define the third-order cumulant

$$\begin{array}{c} K_{ij}\left(\tau_{1},\tau_{3}\right)=\left\langle S_{j}\left(t\right)S_{i}\left(t-\tau_{1}\right)S_{j}\left(t-\tau_{3}\right)\right\rangle -r_{j}(C_{ij}\left(\tau_{1}\right)+C_{ij}\left(\tau_{3}-\tau_{1}\right))-r_{i}C_{jj}\left(\tau_{3}\right)-r_{i}r_{j}^{2}. \end{array}$$ (14)

With these definitions, Eq 11 becomes Eq 1 of the main text.

### Calculation of cumulants

The definition of cumulants in the Fourier space is imperative for the derivation of our results. Assuming stationarity, the expected firing rate (i.e. the first order cumulant) vector ***r*** = 〈**λ**(*t*)〉 no longer depends on time and can be written as

$$\begin{array}{c} r={\left(I-\tilde{E}(0)W\right)}^{-1}\mu , \end{array}$$ (15)

where $\tilde{E}\left(0\right)=F[E(t)]{|}_{t=0}$ denotes the Fourier transform of the EPSC function evaluated at zero. For all the calculations, we define the Fourier transform as

$$\begin{array}{c} F[f(\tau )]=\tilde{f}\left(\omega \right)=\int_{-\infty }^{\infty }f\left(\tau \right)e^{-j\omega \tau }\text{d}\tau . \end{array}$$ (16)

The second-order cumulant, consisting of the cross- and auto-covariance, can be calculated in the time domain as [37, 102]

$$\begin{array}{c} C_{ij}\left(\tau_{1}\right)=\sum_{k=1}^{N}r_{k}\int_{-\infty }^{\infty }R_{ik}\left(u\right)R_{jk}\left(u-\tau_{1}\right)\text{d}u, \end{array}$$ (17)

where $R\left(t\right)=\sum_{n\geq 0}G^{*n}\left(t\right)$ is defined as a ‘convolution power series’ [37, 102] of the interaction kernel ***G***(*t*) = ***W**E*(*t*), with

$$\begin{array}{c} G^{*n}\left(t\right)=\{\begin{array}{cc} I\delta (t), & \text{if}\;n=0\\ \int_{-\infty }^{t}G^{*(n-1)}\left(t-s\right)G\left(s\right)\text{d}s=\int_{-\infty }^{t}{\left(WE\left(t-s\right)\right)}^{*(n-1)}WE\left(s\right)\text{d}s, & \text{if}\;n\geq 1. \end{array} \end{array}$$ (18)

Since ***W*** does not depend on the integration domain, the convolution in the operation *^n^ is calculated on *E*, while for ***W*** it becomes a power operation. Formally, the computation of each element *R*_*mn*_(*t*) consists of calculating the probability of a spike from neuron *m* at time *t* given that neuron *n* fired at time 0. Therefore, in Eq 17 we write the covariance for the spike trains of neurons *i* and *j* as the probability of a pair of spikes in neurons *i* and *j* at a time lag *τ*_1_ given that neuron *k* fired, where *k* can be *any* neuron in the network. This representation provides a convenient formalism for representing causality of spiking events in our model. Then, considering the definition of ‘path lengths’ *α* and *β* from the source neuron *k* to the postsynaptic neuron *i* and the presynaptic neuron *j* (Fig 2A), we can rewrite Eq 17 as

$$\begin{array}{c} C_{ij}\left(\tau_{1}\right)=\sum_{\alpha ,\beta }\int_{-\infty }^{\infty }E^{*\alpha }\left(u\right)E^{*\beta }\left(u-\tau_{1}\right)\text{d}u\sum_{k=1}^{N}r_{k}{\left(W^{\alpha }\right)}_{ik}{\left(W^{\beta }\right)}_{jk}. \end{array}$$ (19)

Here, E*^κ^ denotes a series of convolutions of the EPSC function

$$\begin{array}{c} E^{*\kappa }\left(t\right)=\underset{\kappa \;\text{terms}}{\underset{︸}{E(t)*E(t)*\ldots *E(t)}}. \end{array}$$ (20)

For the auto-covariance *C*_*ii*_ for path lengths *α* and *γ* from the source neuron *k* to the postsynaptic neuron *i* (Fig 2B), we analogously obtain

$$\begin{array}{c} C_{ii}\left(\tau_{2}\right)=\sum_{\alpha ,\gamma }\int_{-\infty }^{\infty }E^{*\alpha }\left(u\right)E^{*\gamma }\left(u-\tau_{2}\right)\text{d}u\sum_{k=1}^{N}r_{k}{\left(W^{\alpha }\right)}_{ik}{\left(W^{\gamma }\right)}_{ik}. \end{array}$$ (21)

Since each *R* function consists of the convolution of the EPSC functions, then its Fourier transform is the product of the Fourier transforms of each of those functions, which simplifies calculations. Therefore, the cross-covariance *C*_*ij*_ in the frequency domain (i.e. the Fourier transform of Eq 17) is given by (detailed derivation in S1 Text)

$$\begin{array}{c} {\tilde{C}}_{ij}\left(\omega \right)=\sum_{k=1}^{N}r_{k}{\tilde{R}}_{ik}\left(\omega \right){\tilde{R}}_{jk}\left(-\omega \right), \end{array}$$ (22)

and, finally we obtain the expression

$$\begin{array}{c} {\tilde{C}}_{ij}\left(\omega \right)=\sum_{\alpha ,\beta }{\tilde{E}}^{\alpha }\left(\omega \right)\;E^{\beta }\left(-\omega \right)\;\sum_{k=1}^{N}r_{k}{\left(W^{\alpha }\right)}_{ik}{\left(W^{\beta }\right)}_{jk}. \end{array}$$ (23)

It should be noted that Eq 23 was also derived in previous works using a different approach [6, 70]. However, for the third-order cumulant *K*_*ij*_ (Fig 4) that same approach is not possible. Therefore, it is convenient to write *K*_*ij*_ in the time domain in terms of the previously defined ***R*** [37, 102] as

$$\begin{array}{c} \begin{array}{cc} K_{ij}\left(\tau_{1},\tau_{2}\right) & =\sum_{k=1}^{N}r_{k}\int_{-\infty }^{\infty }R_{ik}\left(u\right)R_{jk}\left(u-\tau_{1}\right)R_{ik}\left(u-\tau_{2}\right)\text{d}u\\ & +\sum_{k,l=1}^{N}r_{k}\overset{\infty }{\underset{-\infty }{\;\int \int \;}}R_{ik}\left(u\right)R_{jl}\left(v-\tau_{1}\right)R_{il}\left(v-\tau_{2}\right)\Psi_{lk}\left(v-u\right)\text{d}v\;\text{d}u\\ & +\sum_{k,l=1}^{N}r_{k}\overset{\infty }{\underset{-\infty }{\;\int \int \;}}R_{jk}\left(u-\tau_{1}\right)R_{il}\left(v\right)R_{il}\left(v-\tau_{2}\right)\Psi_{lk}\left(v-u\right)\text{d}v\;\text{d}u\\ & +\sum_{k,l=1}^{N}r_{k}\overset{\infty }{\underset{-\infty }{\;\int \int \;}}R_{ik}\left(u-\tau_{2}\right)R_{il}\left(v\right)R_{jl}\left(v-\tau_{1}\right)\Psi_{lk}\left(v-u\right)\text{d}v\;\text{d}u, \end{array} \end{array}$$ (24)

where additionally

$$\begin{array}{c} \Psi \left(t\right)=R\left(t\right)-I\;\delta \left(t\right)=\sum_{n\geq 1}G^{*n}\left(t\right). \end{array}$$ (25)

In Eq 24, Ψ_*lk*_(*v* − *u*) is the probability density of the event that a spike from neuron *k* at a time *v* − *u* = 0 causes a neuron *l* (different from neuron *k*) to emit a spike at a time *v* − *u* ≠ 0, after at least one synaptic connection. The function Ψ is necessary in Eq 24 to take into account the branching structures in the calculation of *K*_*ij*_ (Fig 4B–4D). In addition to *α*, *β* and *γ*, *ζ* is the path length from the source neuron *k* to the neuron *l* where the synaptic connection path branches out and is equal to or larger than one. Then, replacing both the *R* and Ψ functions by their corresponding definitions in terms of the connectivity matrix ***W*** and EPSC function *E*(*t*) yields

$$\begin{array}{c} \begin{array}{cc} K_{ij}\left(\tau_{1},\tau_{2}\right) & =\sum_{\alpha ,\beta ,\gamma }\int_{-\infty }^{\infty }E^{*\alpha }\left(u\right)E^{*\beta }\left(u-\tau_{1}\right)E^{*\gamma }\left(u-\tau_{2}\right)\text{d}u\sum_{k=1}^{N}r_{k}{\left(W^{\alpha }\right)}_{ik}{\left(W^{\gamma }\right)}_{ik}{\left(W^{\beta }\right)}_{jk}\\ & +\sum_{\alpha ,\beta ,\gamma }\sum_{\zeta \geq 1}\overset{\infty }{\underset{-\infty }{\;\int \int \;}}E^{*\alpha }\left(u\right)E^{*\beta }\left(v-\tau_{1}\right)E^{*\gamma }\left(v-\tau_{2}\right)E^{*\zeta }\left(v-u\right)\text{d}v\;\text{d}u\sum_{k,l}^{N}r_{k}{\left(W^{\alpha }\right)}_{ik}{\left(W^{\beta }\right)}_{jl}{\left(W^{\gamma }\right)}_{il}{\left(W^{\zeta }\right)}_{lk}\\ & +\sum_{\alpha ,\beta ,\gamma }\sum_{\zeta \geq 1}\overset{\infty }{\underset{-\infty }{\;\int \int \;}}E^{*\beta }\left(u-\tau_{1}\right)E^{*\alpha }\left(v\right)E^{*\gamma }\left(v-\tau_{2}\right)E^{*\zeta }\left(v-u\right)\text{d}v\;\text{d}u\sum_{k,l}^{N}r_{k}{\left(W^{\beta }\right)}_{jk}{\left(W^{\alpha }\right)}_{il}{\left(W^{\gamma }\right)}_{il}{\left(W^{\zeta }\right)}_{lk}\\ & +\sum_{\alpha ,\beta ,\gamma }\sum_{\zeta \geq 1}\overset{\infty }{\underset{-\infty }{\;\int \int \;}}E^{*\gamma }\left(u-\tau_{2}\right)E^{*\alpha }\left(v\right)E^{*\beta }\left(v-\tau_{1}\right)E^{*\zeta }\left(v-u\right)\text{d}v\;\text{d}u\sum_{k,l}^{N}r_{k}{\left(W^{\gamma }\right)}_{ik}{\left(W^{\alpha }\right)}_{il}{\left(W^{\beta }\right)}_{jl}{\left(W^{\zeta }\right)}_{lk}. \end{array} \end{array}$$ (26)

As with the second-order cumulant, we can calculate the Fourier transform of the third-order cumulant *K*_*ij*_ from Eq 24 as (detailed derivation in S1 Text)

$$\begin{array}{c} \begin{array}{cc} {\tilde{K}}_{ij}\left(\omega_{1},\omega_{2}\right) & =\sum_{k=1}^{N}r_{k}{\tilde{R}}_{ik}\left(\omega_{1}+\omega_{2}\right){\tilde{R}}_{jk}\left(-\omega_{1}\right){\tilde{R}}_{ik}\left(-\omega_{2}\right)\\ & +\sum_{k,l=1}^{N}r_{k}{\tilde{R}}_{ik}\left(\omega_{1}+\omega_{2}\right){\tilde{R}}_{jl}\left(-\omega_{1}\right){\tilde{R}}_{il}\left(-\omega_{2}\right){\tilde{\Psi }}_{lk}\left(\omega_{1}+\omega_{2}\right)\\ & +\sum_{k,l=1}^{N}r_{k}{\tilde{R}}_{il}\left(\omega_{1}+\omega_{2}\right){\tilde{R}}_{jk}\left(-\omega_{1}\right){\tilde{R}}_{il}\left(-\omega_{2}\right){\tilde{\Psi }}_{lk}\left(-\omega_{1}\right)\\ & +\sum_{k,l=1}^{N}r_{k}{\tilde{R}}_{il}\left(\omega_{1}+\omega_{2}\right){\tilde{R}}_{jl}\left(-\omega_{1}\right){\tilde{R}}_{ik}\left(-\omega_{2}\right){\tilde{\Psi }}_{lk}\left(-\omega_{2}\right). \end{array} \end{array}$$ (27)

Finally, we obtain the third-order cumulant *K*_*ij*_ in the Fourier domain in terms of the connectivity matrix ***W***, the EPSC function *E*(*t*), and the path lengths *α*, *β*, *γ* and *ζ* as

$$\begin{array}{c} \begin{array}{cc} K_{ij}\left(\omega_{1},\omega_{2}\right) & =\sum_{\alpha ,\beta ,\gamma }{\tilde{E}}^{\alpha }\left(\omega_{1}+\omega_{2}\right)\;{\tilde{E}}^{\beta }\left(-\omega_{1}\right)\;{\tilde{E}}^{\gamma }\left(-\omega_{2}\right)\;\sum_{k=1}^{N}r_{k}{\left(W^{\alpha }\right)}_{ik}{\left(W^{\gamma }\right)}_{ik}{\left(W^{\beta }\right)}_{jk}\\ & +\sum_{\alpha ,\beta ,\gamma }\sum_{\zeta \geq 1}{\tilde{E}}^{\alpha +\zeta }\left(\omega_{1}+\omega_{2}\right)\;{\tilde{E}}^{\beta }\left(-\omega_{1}\right)\;{\tilde{E}}^{\gamma }\left(-\omega_{2}\right)\;\sum_{k,l=1}^{N}r_{k}{\left(W^{\zeta }\right)}_{lk}{\left(W^{\alpha }\right)}_{ik}{\left(W^{\beta }\right)}_{jl}{\left(W^{\gamma }\right)}_{il}\\ & +\sum_{\alpha ,\beta ,\gamma }\sum_{\zeta \geq 1}{\tilde{E}}^{\alpha }\left(\omega_{1}+\omega_{2}\right)\;{\tilde{E}}^{\beta +\zeta }\left(-\omega_{1}\right){\tilde{E}}^{\gamma }\left(-\omega_{2}\right)\;\sum_{k,l=1}^{N}r_{k}{\left(W^{\zeta }\right)}_{lk}{\left(W^{\alpha }\right)}_{il}{\left(W^{\beta }\right)}_{jk}{\left(W^{\gamma }\right)}_{il}\\ & +\sum_{\alpha ,\beta ,\gamma }\sum_{\zeta \geq 1}{\tilde{E}}^{\alpha }\left(\omega_{1}+\omega_{2}\right)\;{\tilde{E}}^{\beta }\left(-\omega_{1}\right)\;{\tilde{E}}^{\gamma +\zeta }\left(-\omega_{2}\right)\;\sum_{k,l=1}^{N}r_{k}{\left(W^{\zeta }\right)}_{lk}{\left(W^{\alpha }\right)}_{il}{\left(W^{\beta }\right)}_{jl}{\left(W^{\gamma }\right)}_{ik}. \end{array} \end{array}$$ (28)

### Calculation of motif coefficients

Extending the work of [6], who artificially tuned the values of motif coefficients to investigate the consequences on the network structures, we derive them analytically as a function of the STDP rule and the EPSC function. To obtain the expression for the motif coefficients necessary for Eqs 3, 4 and 5, we first need to insert Eqs 23 and 28, i.e. the definitions of the second- and third-order cumulants in the frequency domain, in Eq 2. Then, it can easily be seen that it is possible to separate the part that depends on the products of the connectivity matrix ***W*** from the rest. This way we define the motif coefficients as the integral of the products of the Fourier transforms of the STDP functions and the EPSC functions, considering the appropriate path lengths *α*, *β*, *γ* and *ζ* (Fig 3B and 3D). In particular, for the motif coefficients in Eq 3 we derive

$$\begin{array}{c} M_{\alpha ,\beta }^{pair}=\int_{-\infty }^{\infty }{\tilde{E}}^{\alpha }\left(\omega_{1}\right)\;{\tilde{E}}^{\beta }\left(-\omega_{1}\right)\;{\tilde{L}}_{2}\left(-\omega_{1}\right)\text{d}\omega_{1}. \end{array}$$ (29)

and

$$\begin{array}{c} M_{\alpha ,\beta }^{trip}=\overset{\infty }{\underset{-\infty }{\;\int \int \;}}({\tilde{E}}^{\alpha }\left(\omega_{1}\right)\;{\tilde{E}}^{\beta }\left(-\omega_{1}\right)\;\delta \left(\omega_{2}\right)+{\tilde{E}}^{\alpha }\left(\omega_{2}\right)\;{\tilde{E}}^{\beta }\left(-\omega_{2}\right)\;\delta \left(\omega_{1}+\omega_{2}\right)){\tilde{L}}_{3}\left(-\omega_{1},-\omega_{2}\right)\text{d}\omega_{1}\;\text{d}\omega_{2}. \end{array}$$ (30)

We note that this definition combines motif coefficients where *α* is the index corresponding to paths to the postsynaptic neuron, regardless of which of the two postsynaptic spike of the spike triplet it refers to (Fig 1C). For the motif coefficient in Eq 4 we derive

$$\begin{array}{c} M_{\alpha ,\gamma }^{trip}=\overset{\infty }{\underset{-\infty }{\;\int \int \;}}{\tilde{E}}^{\alpha }\left(\omega_{2}\right)\;{\tilde{E}}^{\gamma }\left(-\omega_{2}\right)\;{\tilde{L}}_{3}\left(-\omega_{1},-\omega_{2}\right)\;\delta \left(\omega_{1}\right)\text{d}\omega_{1}\;\text{d}\omega_{2}. \end{array}$$ (31)

Lastly, for the ‘straight’ triplet motif (Fig 4A) in Eq 5 we get:

$$\begin{array}{c} M_{\alpha ,\beta ,\gamma }^{trip}=\overset{\infty }{\underset{-\infty }{\;\int \int \;}}{\tilde{E}}^{\alpha }\left(\omega_{1}+\omega_{2}\right)\;{\tilde{E}}^{\beta }\left(-\omega_{1}\right)\;{\tilde{E}}^{\gamma }\left(-\omega_{2}\right)\;{\tilde{L}}_{3}\left(-\omega_{1},-\omega_{2}\right)\;\text{d}\omega_{1}\;\text{d}\omega_{2} \end{array}$$ (32)

while for the ‘branching’ motifs (Fig 4B–4D) in Eq 5:

$$\begin{array}{c} M_{(\alpha ,\zeta ),\beta ,\gamma }^{trip}=\overset{\infty }{\underset{-\infty }{\;\int \int \;}}{\tilde{E}}^{\alpha +\zeta }\left(\omega_{1}+\omega_{2}\right)\;{\tilde{E}}^{\beta }\left(-\omega_{1}\right)\;{\tilde{E}}^{\gamma }\left(-\omega_{2}\right)\;{\tilde{L}}_{3}\left(-\omega_{1},-\omega_{2}\right)\;\text{d}\omega_{1}\;\text{d}\omega_{2}, \end{array}$$ (33)

$$\begin{array}{c} M_{\alpha ,(\beta ,\zeta ),\gamma }^{trip}=\overset{\infty }{\underset{-\infty }{\;\int \int \;}}{\tilde{E}}^{\alpha }\left(\omega_{1}+\omega_{2}\right)\;{\tilde{E}}^{\beta +\zeta }\left(-\omega_{1}\right)\;{\tilde{E}}^{\gamma }\left(-\omega_{2}\right)\;{\tilde{L}}_{3}\left(-\omega_{1},-\omega_{2}\right)\;\text{d}\omega_{1}\;\text{d}\omega_{2}, \end{array}$$ (34)

and

$$\begin{array}{c} M_{\alpha ,\beta ,(\gamma ,\zeta )}^{trip}=\overset{\infty }{\underset{-\infty }{\;\int \int \;}}{\tilde{E}}^{\alpha }\left(\omega_{1}+\omega_{2}\right)\;{\tilde{E}}^{\beta }\left(-\omega_{1}\right)\;{\tilde{E}}^{\gamma +\zeta }\left(-\omega_{2}\right)\;{\tilde{L}}_{3}\left(-\omega_{1},-\omega_{2}\right)\;\text{d}\omega_{1}\;\text{d}\omega_{2}. \end{array}$$ (35)

These expressions give us a concise representation of how the spiking activity interacts with network structure to impact plasticity.

### Synaptic dynamics

To calculate the values for the motif coefficients in Eqs 29–35, we define the EPSC function *E*(*t*) as

$$\begin{array}{c} E\left(t\right)=\{\begin{array}{cc} \frac{\tau_{\varepsilon }+\tau_{\iota }}{\tau_{\varepsilon }^{2}}e^{\frac{-t}{\tau_{\varepsilon }}}(1-e^{\frac{-t}{\tau_{\iota }}}) & \text{if}\;t\geq 0\\ 0 & \text{if}\;t<0. \end{array} \end{array}$$ (36)

This function depends on two time constants *τ*_*ε*_ and *τ*_*ι*_ that define the onset and decay of the increase in the membrane potential with each spike. In particular, when *τ*_*ι*_ → 0 the current is instantaneous and decays exponentially. The function is normalized to have an integral equal to 1, so that on average the number of postsynaptic spikes with the arrival of a presynaptic spike scales with the same order of magnitude as the synaptic efficacy. Its Fourier transform is

$$\begin{array}{c} \tilde{E}\left(\omega \right)=(1+\frac{\tau_{\iota }}{\tau_{\varepsilon }})\frac{1-j\tau_{\varepsilon }\omega }{1+\tau_{\varepsilon }^{2}\omega^{2}}-\frac{\tau_{\iota }}{\tau_{\varepsilon }}\frac{1-j\frac{\tau_{\varepsilon }\tau_{\iota }}{\tau_{\varepsilon }+\tau_{\iota }}\omega }{1+{\left(\frac{\tau_{\varepsilon }\tau_{\iota }}{\tau_{\varepsilon }+\tau_{\iota }}\right)}^{2}\omega^{2}}. \end{array}$$ (37)

With respect to the choice of STDP function, we consider the minimal triplet STDP rule [54, 57] that consists of the pair-based STDP function for depression and of a triplet STDP function for potentiation (Fig 1C). Furthermore, we introduce a ‘modulation parameter’ *η*_−_ to model the reshaping of the depression window of the STDP function via modulatory effects.

The depression window of the STDP function can be written as

$$\begin{array}{c} L_{2}\left(\tau_{1}\right)=\{\begin{array}{cc} -\frac{A_{-}}{\eta_{-}}e^{\frac{\tau_{1}}{\eta_{-}\tau_{-}}} & \text{if}\;\tau_{1}<0\\ 0 & \text{otherwise,}\; \end{array} \end{array}$$ (38)

where *τ*_1_ = t_post_ − t_pre_ denotes the time difference between a post- and a presynaptic spike, *A*_−_ is the depression learning rate, *τ*_−_ is the depression time constant and *η*_−_ is the depression modulation parameter. The potentiation window of the STDP function depends on the timing of spike triplets (t_pre_, t_post_, ${\text{t}}_{\text{post}}^{\prime }$)

$$\begin{array}{c} L_{3}\left(\tau_{1},\tau_{2}\right)=\{\begin{array}{cc} A_{+}e^{-\frac{\tau_{1}}{\tau_{+}}}e^{-\frac{\tau_{2}}{\tau_{y}}} & \text{if}\;\tau_{1}\geq 0,\tau_{2}\geq 0\\ 0 & \text{otherwise,} \end{array} \end{array}$$ (39)

where again *τ*_1_ = t_post_ − t_pre_ denotes the time difference between a post- and a presynaptic spike and $\tau_{2}={\text{t}}_{\text{post}}-{\text{t}}_{\text{post}}^{\prime }$ is the time difference between the two postsynaptic spikes; *A*_+_ is the potentiation learning rate, *τ*_+_ is the potentiation time constant and *τ*_*y*_ is the second potentiation time constant.

While the ‘*A*’ parameters scale the amplitude of weight changes, the ‘*τ*’ coefficients determine how synchronous pre- and post-synaptic spikes must be to drive plasticity. The *η*_−_ parameter enables the modification of the shape of the STDP function. This additional parameter does not affect the total depression and potentiation in the rule and one can easily recover the ‘standard’ expressions for *η*_−_ → 1.

The Fourier transforms for these two functions are

$$\begin{array}{c} {\tilde{L}}_{2}\left(\omega_{1}\right)=-A_{-}\tau_{-}\frac{1+j\eta_{-}\tau_{-}\omega_{1}}{1+\eta_{-}^{2}\tau_{-}^{2}\omega_{1}^{2}}, \end{array}$$ (40)

and

$$\begin{array}{c} {\tilde{L}}_{3}\left(\omega_{1},\omega_{2}\right)=A_{+}\tau_{+}\tau_{y}\frac{1-j\tau_{+}\omega_{1}}{1+\tau_{+}^{2}\omega_{1}^{2}}\frac{1-j\tau_{y}\omega_{2}}{1+\tau_{y}^{2}\omega_{2}^{2}}. \end{array}$$ (41)

### Motif expansion up to third-order

After including Eqs 3, 4 and 5 into Eq 2, we can rewrite it in terms of the order of interactions in which they contribute to the averaged synaptic modification:

$$\begin{array}{c} \left\langle {\dot{W}}_{ij}\right\rangle ={\left\langle {\dot{W}}_{ij}\right\rangle }^{\left(0\right)}+{\left\langle {\dot{W}}_{ij}\right\rangle }^{\left(1\right)}+{\left\langle {\dot{W}}_{ij}\right\rangle }^{\left(2\right)}+{\left\langle {\dot{W}}_{ij}\right\rangle }^{\left(3\right)}+\ldots \end{array}$$ (42)

Assuming non-zero mean rates, no self-excitation (i.e. *W*_*ii*_ = 0) and that terms of order higher than three can be disregarded in comparison to lower order ones, the terms of Eq 42 are

$$\begin{array}{c} {\left\langle {\dot{W}}_{ij}\right\rangle }^{\left(0\right)}=r_{i}r_{j}M_{0} \end{array}$$ (43)

for the zeroth-order contributions,

$$\begin{array}{c} {\left\langle {\dot{W}}_{ij}\right\rangle }^{\left(1\right)}=r_{j}W_{ij}M_{1,0}+r_{i}W_{ji}M_{0,1} \end{array}$$ (44)

for the first-order contributions,

$$\begin{array}{c} \begin{array}{cc} {\left\langle {\dot{W}}_{ij}\right\rangle }^{\left(2\right)} & =\sum_{k\neq i,j}r_{k}W_{ik}W_{jk}M_{1,1}+r_{j}{\left(W^{2}\right)}_{ij}M_{2,0}+r_{i}{\left(W^{2}\right)}_{ji}M_{0,2}\\ & +\sum_{k\neq i,j}r_{i}W_{ik}W_{ki}r_{j}M_{\alpha =2,\gamma =0}^{trip}+\sum_{k\neq i,j}r_{k}W_{ik}^{2}r_{j}M_{\alpha =1,\gamma =1}^{trip}\\ & +r_{j}W_{ij}^{2}M_{\alpha =1,\beta =0,\gamma =1}^{trip}, \end{array} \end{array}$$ (45)

for the second-order contributions, and finally

$$\begin{array}{c} \begin{array}{cc} {\left\langle {\dot{W}}_{ij}\right\rangle }^{\left(3\right)} & =\sum_{k\neq i,j}r_{k}{\left(W^{2}\right)}_{ik}W_{jk}M_{2,1}+\sum_{k\neq i,j}r_{k}W_{ik}{\left(W^{2}\right)}_{jk}M_{1,2}+r_{j}{\left(W^{3}\right)}_{ij}M_{3,0}\\ & +r_{i}{\left(W^{3}\right)}_{ji}M_{0,3}+\sum_{k\neq i,j}r_{k}{\left(W^{2}\right)}_{ik}W_{ik}r_{j}(M_{\alpha =2,\gamma =1}^{trip}+M_{\alpha =1,\gamma =2}^{trip})\\ & +r_{j}^{2}{\left(W^{3}\right)}_{ii}M_{\alpha =3,\gamma =0}^{trip}+\sum_{k\neq i,j}r_{k}W_{ik}^{2}W_{jk}M_{\alpha =1,\beta =1,\gamma =1}^{trip} \end{array} \end{array}$$ (46)

for the third-order contributions. Examples and illustrations of these motifs are given in Figs 3 and 5. For conciseness, we grouped motif coefficients arising from the pair-based STDP rule and from the triplet STDP rule that shared values of *α* and *β* and relabeled them as

$$\begin{array}{c} M_{0}={\tilde{L}}_{2}\left(0\right)+r_{i}{\tilde{L}}_{3}\left(0,0\right), \end{array}$$ (47)

$$\begin{array}{c} M_{1,0}=r_{i}M_{\alpha =1,\beta =0}^{trip}, \end{array}$$ (48)

$$\begin{array}{c} M_{0,1}=M_{\alpha =0,\beta =1}^{pair}+r_{i}M_{\alpha =0,\beta =1}^{trip}, \end{array}$$ (49)

$$\begin{array}{c} M_{2,0}=r_{i}M_{\alpha =2,\beta =0}^{trip}, \end{array}$$ (50)

$$\begin{array}{c} M_{0,2}=M_{\alpha =0,\beta =2}^{pair}+r_{i}M_{\alpha =0,\beta =2}^{trip}, \end{array}$$ (51)

$$\begin{array}{c} M_{1,1}=M_{\alpha =1,\beta =1}^{pair}+r_{i}M_{\alpha =1,\beta =1}^{trip}, \end{array}$$ (52)

$$\begin{array}{c} M_{3,0}=r_{i}M_{\alpha =3,\beta =0}^{trip}, \end{array}$$ (53)

$$\begin{array}{c} M_{0,3}=M_{\alpha =0,\beta =3}^{pair}+r_{i}M_{\alpha =0,\beta =3}^{trip}, \end{array}$$ (54)

$$\begin{array}{c} M_{2,1}=M_{\alpha =2,\beta =1}^{pair}+r_{i}M_{\alpha =2,\beta =1}^{trip}, \end{array}$$ (55)

$$\begin{array}{c} M_{1,2}=M_{\alpha =1,\beta =2}^{pair}+r_{i}M_{\alpha =1,\beta =2}^{trip}. \end{array}$$ (56)

Using the defined functions for the EPSC (Eq 37) and STDP functions (Eqs 40 and 41) we calculated the motif coefficients in Eqs 29–32, and consequently Eqs 47–56. Note, we excluded the higher-than-third-order motif coefficients in Eqs 33–35. The truncated approximation of motifs up to third-order is valid, since the difference of the weight change from the full contribution and the weight change from up to third-order motif truncation is very small (S1 Fig). These quantities represent the strength of contributions of each particular combination of paths from the source neuron to the pre- and postsynaptic neurons involved in the synaptic connection. In principle, also the motifs *M*_*α*=0,*γ*=2_ and *M*_*α*=0,*γ*=3_ from the auto-covariance and the motifs *M*_*α*=2,*β*=0,*γ*=1_ and *M*_*α*=1,*β*=0,*γ*=2_ from the third-order cumulant would need to be considered, however we find that the contribution of these motifs is zero and therefore we did not include them in our analysis. Since we assume a learning rule balanced in potentiation and depression,

$$\begin{array}{c} M_{0}=0, \end{array}$$ (57)

and thus

$$\begin{array}{c} -A_{-}\tau_{-}+r_{i}A_{+}\tau_{+}\tau_{y}=0, \end{array}$$ (58)

which is independent of the modulation parameter *η*_−_, this allows us to rewrite these motifs so that they are independent of the mean firing rate of the postsynaptic neuron. Since the firing rate *r*_*i*_ is not fixed, it should be noted that this assumption implies that the amplitude of the LTP window *A*_+_ adjusts to balance the learning rule, similar to metaplasticity [57]. However, we verify that the firing rates in the system are relatively stable over the time of the simulation (S5 Fig) and therefore *A*_+_ does not vary much. We analyze the evolution of these quantities in the main text, because they involve only *α* and *β* paths and remain constant throughout the numerical integration, in contrast to the motif coefficients in Eqs 45 and 46 which involve both *α* and *γ* paths, and which have an additional rate dependence. The expressions for the motif coefficients defined by Eqs 47–56 in terms of the EPSC and STDP functions’ parameters are given in S2 Text.

### Perturbation of the zero-order motif

We consider a small perturbation *δ* to the zero-order (or rate) motif

$$\begin{array}{c} M_{0}=\pm \delta . \end{array}$$ (59)

A minus sign indicates that the balance is tilted towards depression and, conversely, a plus sign conveys a potentiation-dominated regime. Then, given the minimal triplet STDP rule, we obtain that

$$\begin{array}{c} -A_{-}\tau_{-}+r_{i}A_{+}\tau_{+}\tau_{y}=\pm \delta . \end{array}$$ (60)

Since the firing rate contribution to plasticity is now different from zero, chance spike coincidences impact the averaged evolution of the synaptic efficacies as follows

$$\begin{array}{c} \left\langle {\dot{W}}_{ij}\right\rangle =r_{i}r_{j}\left(\pm \delta \right)+\cdots \end{array}$$ (61)

In this new scenario, the motif coefficients calculated from the triplet rule (equations given in S2 Text) now scale with (*A*_−_ *τ*_−_ ± *δ*). All weights depress to zero for *δ* < 0 because we impose a lower bound on the weights at 0, and do not include any growth terms independent of synaptic potentiation.

### Additional plasticity mechanisms besides STDP

Although we did not formally model inhibitory plasticity, we assume that the overall effect of the inhibitory population on the synaptic efficacies among excitatory neurons is to balance the network activity. Thus, the sum of inhibitory synapses into each neuron is dynamically adjusted to match the sum of the excitatory synaptic efficacies, such that each element of the inhibitory connectivity matrix is equal to the average of the excitatory input as

$$\begin{array}{c} W^{\text{inh}}=[\begin{array}{c} w_{1}^{\text{inh}}\\ \vdots \\ w_{N}^{\text{inh}} \end{array}]\left[1\cdots 1\right]-D^{\text{inh}} \end{array}$$ (62)

where

$$\begin{array}{c} w_{l}^{\text{inh}}=\frac{1}{N-1}\sum_{k}W_{lk} \end{array}$$ (63)

is the value of each row element and

$$\begin{array}{c} D^{\text{inh}}=[\begin{array}{ccc} w_{1}^{\text{inh}} & \cdots & 0\\ 0 & \ddots & 0\\ 0 & \cdots & w_{N}^{\text{inh}} \end{array}] \end{array}$$ (64)

is a diagonal matrix to take into account there is no self-connectivity. Then, the effective connectivity weight matrix is calculated as ***W***^eff^ = ***W*** − ***W***^inh^. The inhibitory connections are fast and updated in each integration step. As mentioned earlier, we refer to ***W***^eff^ as ***W*** in the manuscript. It should be noted that deviations from this perfect balance between excitation and inhibition, modeled with an inhibitory multiplicative factor *δ*_*inh*_ which scales the overall inhibitory inputs to deviate from a perfect balance, do not affect the emergence of network structure (S4 Fig). Furthermore, we find that the formation of network structure does somewhat depend on the input rate *μ*_*i*_ but not on heterogeneity in the input firing rates (S3 Fig). The input rates effectively determine the mean firing rates of the network throughout the whole simulation (S5 Fig).

We also implement heterosynaptic competition based on previous work [6, 80] as an additional mechanism for the plasticity dynamics to restrict the maximum number of strong connections a neuron can make, and thus keep the spectral radius of the connectivity matrix lower than one. The total synaptic input and output of each neuron is limited: the sum of the inbound (afferent) connections to each postsynaptic neuron *i* and the sum of outbound (efferent) excitatory synaptic efficacies from each presynaptic neuron *j* have an upper bound *W*_max_. The plasticity due to heterosynaptic competition can be written as

$$\begin{array}{c} \left\langle {\dot{W}}_{ij}^{\text{hc}}\right\rangle =(W_{\text{max}}-\sum_{k}W_{ki})H(\sum_{k}W_{ki}-W_{\text{max}})+(W_{\text{max}}-\sum_{k}W_{jk})H(\sum_{k}W_{jk}-W_{\text{max}}), \end{array}$$ (65)

where *H* is the Heaviside function. Imposing an upper bound *w*_max_ for each synaptic efficacy restricts the possible number of connections a neuron can make to $\frac{W_{\text{max}}}{w_{\text{max}}}$. Therefore, the average amount of plasticity is the sum of the change due to STDP based on Eq 2 and heterosynaptic competition based on Eq 65.

$$\begin{array}{c} \left\langle {\dot{W}}_{ij}\right\rangle =\nu (\left\langle {\dot{W}}_{ij}^{\text{STDP}}\right\rangle +\psi \left\langle {\dot{W}}_{ij}^{\text{hc}}\right\rangle ). \end{array}$$ (66)

Here, the learning rate scale *ν* ensures that the synaptic efficacy increments in each integration step are small. The relative contribution of heterosynaptic competition to overall plasticity is determined by the heterosynaptic competition term *ψ*. The values for these parameters can be found in Table 1. The values for the parameters in the Supplementary Figures can be found in Table 2.

**Table 2:** Parameter values for supplementary figures. ⋆ denotes that values are provided in the figures.

Symbol	Description	S1 Fig	S2 Fig	S3 Fig	S4 Fig	S5 Fig
*N*	Number of neurons	12	48			
***μ***	External input firing rate	150 Hz		⋆	150 Hz	⋆
*w*_*max*_	Upper bound for each individual weight	0.17				
*W*_*max*_	Upper bound for total row/ column weight	0.85				
*A*_−_	Depression learning rate	0.01				
*τ*_−_	Depression time constant	33.7 ms [54]				
*τ*_+_	Potentiation time constant	16.8 ms [54]				
*τ*_*y*_	Second potentiation time constant	114 ms [54]				
*η*_−_	Depression modulation parameter	13	⋆	13		
*τ*_*ε*_	First membrane time constant	5 ms				
*τ*_*ι*_	Second membrane time constant	5 ms				
*ν*	Scaling parameter of learning rate	3.5 ×10^−4^				
*ψ*	Heterosynaptic competition scaling parameter	0.7				

### Numerical integration of connectivity matrices

To generate the different connectivity matrices in each Figure, we integrate Eq 2 numerically. The plasticity dynamics are implemented using the Euler method with an adaptive time step. The maximal amount that a weight can change in each integration step is 0.00035. Although the weight evolution is deterministic and determined by the plasticity parameters (Table 1), final connectivity matrices depend on initial connectivity matrices. The initial connection weights are chosen independently from a uniform distribution between 0 and *W*_*max*_/*N* × 0.001, and each one of these initial conditions corresponds to a different “trial”. The numerical integration for each initial condition is continued until the network connectivity achieves a steady state (no longer changes).

### Averaged ordered connectivity matrices

The connectivity matrices resulting from integrating Eq 2 numerically are ordered to reflect the graph structure of the network [6] (Fig 6C). K-means classification groups neurons that share similar connectivity using a squared Euclidean distance. We then reorder the connectivity matrix based on the groups identified by the k-means clustering. Since the structures studied depend on initial conditions, despite the deterministic nature of our approach, we average the rearranged synaptic efficacy matrix over many trials with different (but random and weak) initial connectivity to obtain the most likely connectivity (Figs 6E and 9C). Assemblies on the edges of the connectivity matrices have sharper edges due to an artifact created by the ordering algorithm, but this does not affect results.

### Network analysis

We calculated graph theoretic measures for directed networks using algorithms of the Brain Connectivity Toolbox [124] from http://www.brain-connectivity-toolbox.net. All graph measures were calculated at the steady state and increased during a simulation as network organization improved.

#### Clustering coefficient

For each connectivity matrix we computed the clustering coefficient [95]. For node *i*, this is

$$\begin{array}{c} C_{i}=\frac{\text{number}\;\text{of}\;\text{complete}\;\text{triplets}}{\text{number}\;\text{of}\;\text{all}\;\text{possible}\;\text{triplets}}. \end{array}$$ (67)

The number of complete triplets is obtained from the product of the corresponding edges of the node (from the adjancency matrix), and the total number of triplets depends on network size. Then, the average of the clustering coefficients of all the vertices *N* is given by [92]

$$\begin{array}{c} \bar{C}=\frac{1}{N}\sum_{i=1}^{N}C_{i}. \end{array}$$ (68)

#### Global efficiency

The efficiency in the communication between nodes *i* and *j* can be defined to be inversely proportional to the shortest distance. The average efficiency of a network is calculated as [96]

$$\begin{array}{c} E=\frac{1}{N(N-1)}\sum_{i\neq j}\frac{1}{d(i,j)} \end{array}$$ (69)

where *N* denotes the nodes in the network and *d*(*i*, *j*) is the length of the shortest path between a node *i* and a different node *j*. As an alternative to the average path length, the global efficiency of a network is defined as

$$\begin{array}{c} E_{glob}=\frac{E}{E(\text{full}\;\text{network})} \end{array}$$ (70)

where the efficiency is scaled by an ideal graph where all the possible edges exist (i.e. full network). The difference between these measures is that the first measure quantifies the efficiency in a network where only one packet of information is being moved through it and the global measure quantifies the efficiency where all the vertices are exchanging packets of information with each other [96].

#### Modularity

The modularity *Q* of a connectivity matrix is a measure of the strength of its division into clusters or modules. Formally, modularity can be calculated as [93]

$$\begin{array}{c} Q=\frac{1}{2m}\sum_{vw}(A_{vw}-\frac{k_{v}k_{w}}{2m})\frac{s_{v}s_{w}+1}{2} \end{array}$$ (71)

where ***A*** is the adjancency matrix of the graph, *k* is the node degree, *v* and *w* are the nodes’ indices and *s* is a variable that determines if the node belongs to a community or not. Modularity is the non-randomly distributed proportion of the edges that belong to the given cluster in a graph. It is positive if the number of edges within groups exceeds the number expected at random and depends on the chosen method for community detection.

The first algorithm we used for community detection, referred to as ‘spectral clustering’ algorithm, is based on the fact that modularity of a network is closely related to the structure of the eigenvalue spectrum of its weight matrix [93, 125, 126], high modularity means more strongly embedded communities. This is reflected in the spectra of the connectivity matrices as the separation of eigenvalues into a group with most eigenvalues and another of outliers, the number of which is often used to estimate the number of communities present in the network [93, 125, 126].

The second algorithm, called the Louvain method [98, 124] is a greedy optimization method. First, smaller cliques are found by optimizing modularity locally on all nodes, then each small-sized community is grouped into one node and the first step is repeated. The complete modularity is then calculated by maximizing this value over all the divisions of the network into clusters [98, 124]. We did not find any relevant differences between the Louvain method [98] and the spectral clustering algorithm [93, 125, 126], which were used to define community structure (see Fig 7C).

## Supporting information

S1 Fig The truncated weight change including motifs up to third-order provides a good match for the full weight change including motifs of all orders. The total weight change is calculated either by including motifs up to third-order, or using the full contribution of all motifs based on the integral of Eq 2 (main text) in Fourier space. Each dot represents the respective weight change calculated with the truncated (abscissa) and the full version (ordinate) starting with a random set of initial connectivity weights. The axis are normalized to the maximum weight change. Parameters used are the ones used in the manuscript, except for *N* = 12 (to speed up calculations) and *η*_−_ = 13.
(TIF)

S2 Fig Spontaneous emergence of assemblies does not depend on the third-order cumulant. Considering only (up to third order) motifs related to the second-order cross-covariance *C*_*ij*_ (blue) leads to generally worse graph measures compared to the case when adding motifs from the second-order auto-covariance *C*_*ii*_ (red) and the case where all motifs are considered (yellow). **A**. Mean clustering coefficient versus the modulation parameter *η*_−_. **B**. Mean global efficiency versus the modulation parameter *η*_−_. **C**. Mean modularity versus the modulation parameter *η*_−_. All results are calculated from 100 trials at steady state connectivity. Error bars represent the standard error of the mean.
(TIF)

S3 Fig Network structure at steady state is sensitive to the external input firing rate, but not to heterogeneities in the input firing rates. **A**. Mean clustering coefficient, mean global efficiency and modularity versus the external input firing rate. Assembly formation breaks down for very large input firing rates. **B**. Mean clustering coefficient, mean global efficiency and modularity versus the standard deviation of firing rate distribution, *σ*, to introduce heterogeneity in the external input firing rates. Varying *σ* preserves assembly formation as can be seen from the different graph measures. The mean external input rate was chosen to be 150 Hz. The modulation parameter used is *η*_−_ = 13 and all other parameters are taken as in the main text. Note that the abscissa is logarithmic.
(TIF)

S4 Fig Departures from the balance of excitation and inhibition do not effect assembly formation. Mean clustering coefficient, mean global efficiency and modularity versus the inhibitory multiplicative factor *δ*_inh_ = {0.5, 0.8, 1.2, 1.5}, which scales the overall inhibitory matrix (see Methods). Increasing or decreasing *δ*_inh_ does not disrupt assembly formation as can be seen from the comparison of the different graph measures. The modulation parameter used is *η*_−_ = 13 and all other parameters are taken as in the main text.
(TIF)

S5 Fig The network converges to steady firing rates during ongoing plasticity. The different curves indicate different external input firing rates and in each case the network converges to the same rate as the external input firing rate. The modulation parameter used is *η*_−_ = 13 and all other parameters are taken as in the main text.
(TIF)

S1 Text Fourier transform of the second- and third-order cumulants. (PDF)

S2 Text Calculation of motif coefficients up to third-order. (PDF)
