# Supplementary figures and images for: Autonomous emergence of connectivity assemblies via spike triplet interactions
Source: PLoS Comput Biol. 2020 May 8;16(5):e1007835. doi: 10.1371/journal.pcbi.1007835 (PMC7239496; doi:10.1371/journal.pcbi.1007835)

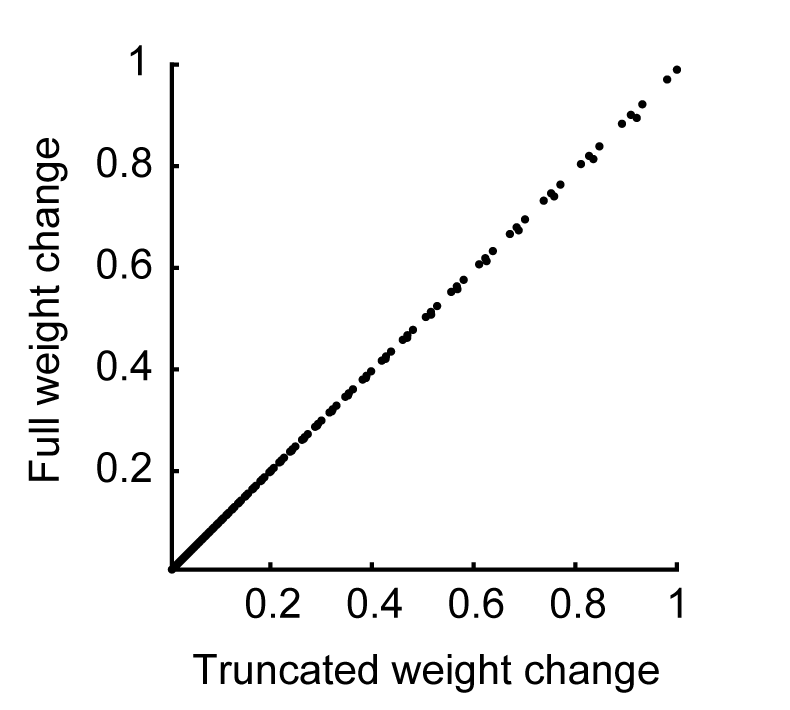

Supplement: S1 Fig — The total weight change is calculated either by including motifs up to third-order, or using the full contribution of all motifs based on the integral of Eq 2 (main text) in Fourier space. Each dot represents the respective weight change calculated with the truncated (abscissa) and the full version (ordinate) starting with a random set of initial connectivity weights. The axis are normalized to the maximum weight change. Parameters used are the ones used in the manuscript, except for N = 12 (to speed up calculations) and η− = 13. (TIF) [file pcbi.1007835.s001.tif]

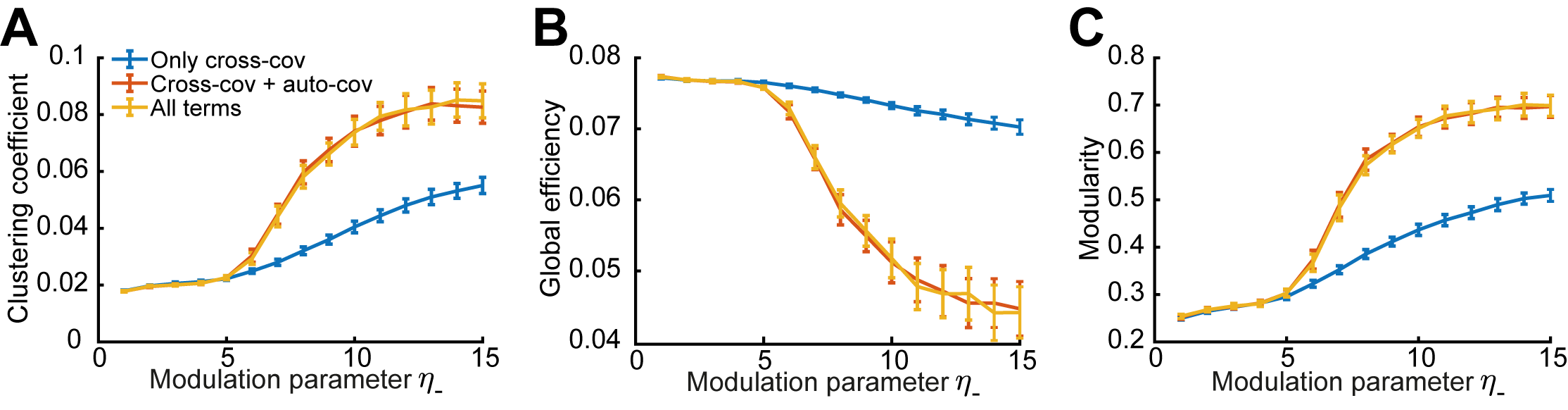

Supplement: S2 Fig — Considering only (up to third order) motifs related to the second-order cross-covariance Cij (blue) leads to generally worse graph measures compared to the case when adding motifs from the second-order auto-covariance Cii (red) and the case where all motifs are considered (yellow). A. Mean clustering coefficient versus the modulation parameter η−. B. Mean global efficiency versus the modulation parameter η−. C. Mean modularity versus the modulation parameter η−. All results are calculated from 100 trials at steady state connectivity. Error bars represent the standard error of the mean. (TIF) [file pcbi.1007835.s002.tif]

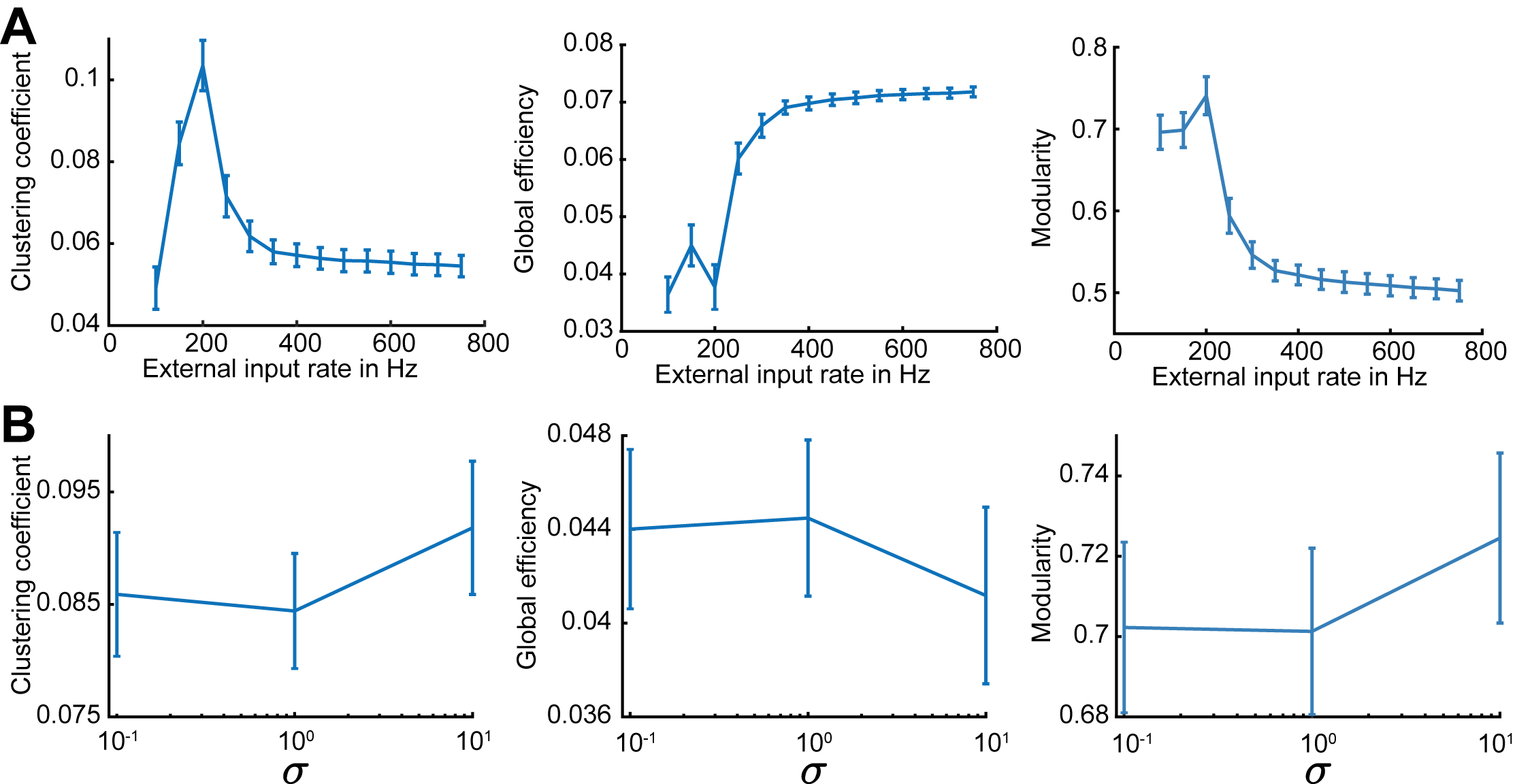

Supplement: S3 Fig — A. Mean clustering coefficient, mean global efficiency and modularity versus the external input firing rate. Assembly formation breaks down for very large input firing rates. B. Mean clustering coefficient, mean global efficiency and modularity versus the standard deviation of firing rate distribution, σ, to introduce heterogeneity in the external input firing rates. Varying σ preserves assembly formation as can be seen from the different graph measures. The mean external input rate was chosen to be 150 Hz. The modulation parameter used is η− = 13 and all other parameters are taken as in the main text. Note that the abscissa is logarithmic. (TIF) [file pcbi.1007835.s003.tif]

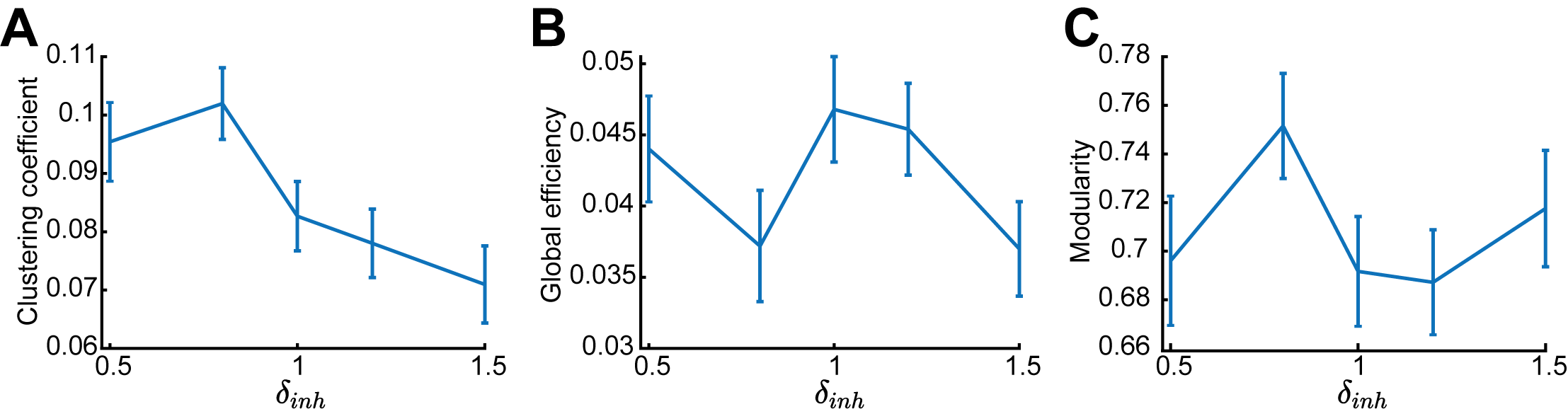

Supplement: S4 Fig — Mean clustering coefficient, mean global efficiency and modularity versus the inhibitory multiplicative factor δinh = {0.5, 0.8, 1.2, 1.5}, which scales the overall inhibitory matrix (see Methods). Increasing or decreasing δinh does not disrupt assembly formation as can be seen from the comparison of the different graph measures. The modulation parameter used is η− = 13 and all other parameters are taken as in the main text. (TIF) [file pcbi.1007835.s004.tif]

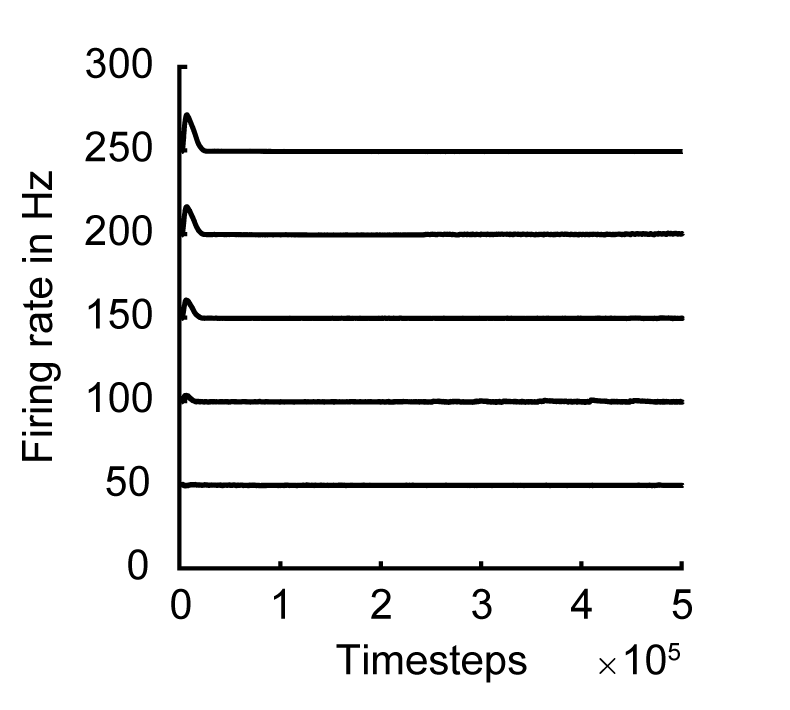

Supplement: S5 Fig — The different curves indicate different external input firing rates and in each case the network converges to the same rate as the external input firing rate. The modulation parameter used is η− = 13 and all other parameters are taken as in the main text. (TIF) [file pcbi.1007835.s005.tif]
