# Supplementary material for: Autonomous emergence of connectivity assemblies via spike triplet interactions
Source: PLoS Comput Biol. 2020 May 8;16(5):e1007835. doi: 10.1371/journal.pcbi.1007835 (PMC7239496; doi:10.1371/journal.pcbi.1007835)
Supplement: S1 Text — (PDF) [file pcbi.1007835.s006.pdf]

S1 Text

Autonomous emergence of connectivity assemblies via spike triplet interactions

Lisandro Montangie[1], Christoph Miehl[1,2], Julijana Gjorgjieva[1,2*],

1 Max Planck Institute for Brain Research, 60438 Frankfurt, Germany
2 Technical University of Munich, School of Life Sciences, 85354 Freising, Germany

*gjorgjieva@brain.mpg.de

Fourier transform of covariance

We calculate the Fourier transform of the covariance C_{ij} (Eq. 29 in the Methods section) as

$$\begin{aligned}\tilde{C}_{ij}(\omega) &= \int\limits_{-\infty}^{\infty} \left(\sum_{k=1}^{N} r_k \int_{-\infty}^{\infty} R_{ik}(u) R_{jk}(u-\tau) du \right) e^{-j\omega\tau} d\tau \\ &= \sum_{k=1}^{N} r_k \int\limits_{-\infty}^{\infty} R_{ik}(u) \left(\int_{-\infty}^{\infty} R_{jk}(u-\tau) e^{-j\omega\tau} d\tau \right) du.\end{aligned} \tag{S1.1}$$

Multiplying the right side of the equation by $e^{\pm j\omega u}$ and changing the variables as

$$\begin{cases} \tau &= x + y \\ u &= y \end{cases} \tag{S1.2}$$

Then, since the Jacobian of the transformation is 1, we obtain Eq. 33 as

$$\begin{aligned}\tilde{C}_{ij}(\omega) &= \sum_{k=1}^{N} r_k \int\limits_{-\infty}^{\infty} R_{ik}(y) e^{-j\omega y} \left(\int_{-\infty}^{\infty} R_{jk}(-x) e^{-jx\omega} dx \right) dy \\ &= \sum_{k=1}^{N} r_k \tilde{R}_{jk}(-\omega) \int\limits_{-\infty}^{\infty} R_{ik}(y) e^{-j\omega y} dy. \\ &= \sum_{k=1}^{N} r_k \tilde{R}_{ik}(\omega) \tilde{R}_{jk}(-\omega).\end{aligned} \tag{S1.3}$$

Fourier transform of third order cumulant

In the same manner, we calculate the Fourier transform of the third order cumulant K_{ij} (Eq. 35 in the Methods section) as

$$\begin{aligned}\tilde{K}_{ij}(\omega_1,\omega_2) = \iint_{-\infty}^{\infty} d\tau_1 d\tau_2 e^{-j(\omega_1\tau_1+\omega_2\tau_2)} \Bigg(\sum_{k=1}^{N} r_k \int_{-\infty}^{\infty} R_{ik}(u)R_{jk}(u-\tau_1)R_{ik}(u-\tau_2)du \\ + \sum_{k,l=1}^{N} r_k \iint_{-\infty}^{\infty} R_{ik}(u)R_{jl}(v-\tau_1)R_{il}(v-\tau_2)\Psi_{lk}(v-u)dvdu \\ + \sum_{k,l=1}^{N} r_k \iint_{-\infty}^{\infty} R_{jk}(u-\tau_1)R_{il}(v)R_{il}(v-\tau_2)\Psi_{lk}(v-u)dvdu \\ + \sum_{k,l=1}^{N} r_k \iint_{-\infty}^{\infty} R_{ik}(u-\tau_2)R_{il}(v)R_{jl}(v-\tau_1)\Psi_{lk}(v-u)dvdu \Bigg).\end{aligned} \tag{S1.4}$$

Although Eq. S1.4 consists on four different terms, since the Fourier transform is a linear operation we can calculate each term independently. The first term is given by

$$\begin{aligned}(1) &= \sum_{k=1}^{N} r_k \iiint_{-\infty}^{\infty} R_{ik}(u)R_{jk}(u-\tau_1)R_{ik}(u-\tau_2)e^{-j(\omega_1\tau_1+\omega_2\tau_2)}d\tau_1 d\tau_2 du \\ &= \sum_{k=1}^{N} r_k \int_{-\infty}^{\infty} R_{ik}(u) \int_{-\infty}^{\infty} R_{jk}(u-\tau_1)e^{-j\omega_1\tau_1}d\tau_1 \int_{-\infty}^{\infty} R_{ik}(u-\tau_2)e^{-j\omega_2\tau_2}d\tau_2 du.\end{aligned} \tag{S1.5}$$

Multiplying the right side of the equation by $e^{\pm j\omega_1 u}$ and $e^{\pm j\omega_2 u}$ and changing the variables as

$$\begin{cases} \tau_1 &= x+y \\ \tau_2 &= x'+y \\ u &= y \end{cases} \tag{S1.6}$$

Again, since the Jacobian of the transformation is 1, we obtain

$$\begin{aligned}(1) &= \sum_{k=1}^{N} r_k \int_{-\infty}^{\infty} R_{ik}(y)e^{-j(\omega_1+\omega_2)y}dy \int_{-\infty}^{\infty} R_{jk}(-x)e^{-j\omega_1 x}dx \int_{-\infty}^{\infty} R_{ik}(-x')e^{-j\omega_2 x'}dx' \\ &= \sum_{k=1}^{N} r_k \tilde{R}_{ik}(\omega_1+\omega_2)\tilde{R}_{jk}(-\omega_1)\tilde{R}_{ik}(-\omega_2).\end{aligned} \tag{S1.7}$$

The second term of Eq. S1.4 is

$$\begin{aligned}(2) &= \sum_{k,l=1}^{N} r_k \iiiint_{-\infty}^{\infty} R_{ik}(u)R_{jl}(v-\tau_1)R_{il}(v-\tau_2)\Psi_{lk}(v-u)e^{-j(\omega_1\tau_1+\omega_2\tau_2)}dvdud\tau_1 d\tau_2 \\ &= \sum_{k,l=1}^{N} r_k \iint_{-\infty}^{\infty} R_{ik}(u)\Psi_{lk}(v-u) \int_{-\infty}^{\infty} R_{jl}(v-\tau_1)e^{-j\omega_1\tau_1}d\tau_1 \int_{-\infty}^{\infty} R_{il}(v-\tau_2)e^{-j\omega_2\tau_2}d\tau_2 dvdu\end{aligned} \tag{S1.8}$$

First, we multiply the right side of the equation by $e^{\pm j\omega_1 v}$ and $e^{\pm j\omega_2 v}$ and change the variables as

$$\begin{cases} \tau_1 &= x + y' \\ \tau_2 &= x' + y' \\ u &= y \\ v &= y' \end{cases} \tag{S1.9}$$

Again, since the Jacobian of the transformation is 1, we obtain

$$\begin{aligned} (2) &= \sum_{k,l=1}^{N} r_k \iint\limits_{-\infty}^{\infty} R_{ik}(y)\Psi_{lk}(y'-y)e^{-j(\omega_1+\omega_2)y'}dy'dy \int\limits_{-\infty}^{\infty} R_{jl}(-x)e^{-j\omega_1 x}dx \int\limits_{-\infty}^{\infty} R_{il}(-x')e^{-j\omega_2 x'}d\tau_2 \\ &= \sum_{k,l=1}^{N} r_k \tilde{R}_{jl}(-\omega_1)\tilde{R}_{il}(-\omega_2) \iint\limits_{-\infty}^{\infty} R_{ik}(y)\Psi_{lk}(y'-y)e^{-j(\omega_1+\omega_2)y'}dy'dy. \end{aligned} \tag{S1.10}$$

Second, we multiply the right side of the equation by $e^{\pm j(\omega_1+\omega_2)y}$ and change the variables as

$$\begin{cases} y' &= x'' + y'' \\ y &= y'' \end{cases} \tag{S1.11}$$

Next, the second term now is calculated as

$$\begin{aligned} (2) &= \sum_{k,l=1}^{N} r_k \tilde{R}_{il}(-\omega_1)\tilde{R}_{jl}(-\omega_2) \int\limits_{-\infty}^{\infty} R_{ik}(y'')e^{-j(\omega_1+\omega_2)y''}dy'' \int\limits_{-\infty}^{\infty} \Psi_{lk}(x'')e^{-j(\omega_1+\omega_2)x''}dx'' \\ &= \sum_{k,l=1}^{N} r_k \tilde{R}_{ik}(\omega_1+\omega_2)\tilde{R}_{jl}(-\omega_1)\tilde{R}_{il}(-\omega_2)\tilde{\Psi}_{lk}(\omega_1+\omega_2). \end{aligned} \tag{S1.12}$$

The last two terms (3) and (4) of Eq. S1.4 are solved in the same way as done with the second term, and we obtain

$$(3) = \sum_{k,l=1}^{N} r_k \tilde{R}_{il}(\omega_1+\omega_2)\tilde{R}_{jk}(-\omega_1)\tilde{R}_{il}(-\omega_2)\tilde{\Psi}_{lk}(-\omega_1) \tag{S1.13}$$

and

$$(4) = \sum_{k,l=1}^{N} r_k \tilde{R}_{il}(\omega_1+\omega_2)\tilde{R}_{jl}(-\omega_1)\tilde{R}_{ik}(-\omega_2)\tilde{\Psi}_{lk}(-\omega_2). \tag{S1.14}$$

Finally, we derive Eq. 38 by summing all four terms in the Fourier domain.
