# Supplementary material for: Autonomous emergence of connectivity assemblies via spike triplet interactions
Source: PLoS Comput Biol. 2020 May 8;16(5):e1007835. doi: 10.1371/journal.pcbi.1007835 (PMC7239496; doi:10.1371/journal.pcbi.1007835)
Supplement: S2 Text — (PDF) [file pcbi.1007835.s007.pdf]

S2 Text

Autonomous emergence of connectivity assemblies via spike triplet interactions

Lisandro Montangie[1], Christoph Miehl[1,2], Julijana Gjorgjieva[1,2*],

1 Max Planck Institute for Brain Research, 60438 Frankfurt, Germany
2 Technical University of Munich, School of Life Sciences, 85354 Freising, Germany

*gjorgjieva@brain.mpg.de

Calculation of motif coefficients up to third order

Under the condition that the STDP rule is balanced, in the sense that the total depression is equal to the total potention, we obtain the motif coefficients for the motif expansion up to third order in terms of the parameters of the STDP and EPSP functions. The first order motif coefficients that involve α- and β-paths can be calculated as

$$M_{1,0} = 2\pi A_-\tau_-\left(\frac{\tau_+}{(\tau_\varepsilon+\tau_+)(\tau_\chi+\tau_+)} + \frac{\tau_y^2}{(\tau_\varepsilon+\tau_y)(\tau_++\tau_y)(\tau_\chi+\tau_y)}\right), \tag{S2.1}$$

and

$$M_{0,1} = -2\pi A_-\tau_-\left(\frac{\eta_-\tau_-}{(\tau_\varepsilon+\eta_-\tau_-)(\tau_\chi+\eta_-\tau_-)} - \frac{\tau_+^2}{(\tau_\varepsilon+\tau_+)(\tau_++\tau_y)(\tau_\chi+\tau_+)}\right), \tag{S2.2}$$

where $\tau_\chi = \frac{\tau_\varepsilon\tau_\iota}{\tau_\varepsilon+\tau_\iota}$. The second order motif coefficients are

$$M_{2,0} = 2\pi A_-\tau_-\left(\frac{\tau_+^3}{(\tau_\varepsilon+\tau_+)^2(\tau_\chi+\tau_+)^2} + \frac{\tau_y^4}{(\tau_\varepsilon+\tau_y)^2(\tau_++\tau_y)(\tau_\chi+\tau_y)^2}\right), \tag{S2.3}$$

$$M_{0,2} = -2\pi A_-\tau_-\left(\frac{\eta_-^3\tau_-^3}{(\tau_\varepsilon+\eta_-\tau_-)^2(\tau_\chi+\eta_-\tau_-)^2} - \frac{\tau_+^4}{(\tau_\varepsilon+\tau_+)^2(\tau_++\tau_y)(\tau_\chi+\tau_+)^2}\right), \tag{S2.4}$$

and

$$\begin{aligned} M_{1,1} = \pi A_-\tau_-\Bigg(&\frac{(\tau_++\tau_\upsilon)}{(\tau_\varepsilon+\tau_+)(\tau_++\tau_\chi)} + \frac{2\tau_+^4}{(\tau_++\tau_y)(\tau_+^2-\tau_\varepsilon^2)(\tau_+^2-\tau_\chi^2)} + \frac{(\tau_\varepsilon+\tau_\iota)^2}{(\tau_\varepsilon+2\tau_\iota)(\tau_\varepsilon-\tau_+)(\tau_\varepsilon+\tau_y)} \\ &+ \frac{\tau_\iota^3}{(\tau_\varepsilon+2\tau_\iota)(\tau_\varepsilon+\tau_\iota)(\tau_+-\tau_\chi)(\tau_y+\tau_\chi)} - \frac{(\eta_-\tau_-+\tau_\upsilon)}{(\tau_\varepsilon+\eta_-\tau_-)(\eta_-\tau_-+\tau_\chi)}\Bigg), \end{aligned} \tag{S2.5}$$

where $\tau_\upsilon = \frac{\tau_\varepsilon \tau_\iota}{\tau_\varepsilon + 2\tau_\iota}$. Finally, the four third order coefficients are calculated as

$$M_{3,0} = 2\pi A_- \tau_- \left(\frac{\tau_+^5}{(\tau_\varepsilon + \tau_+)^3 (\tau_\chi + \tau_+)^3} + \frac{\tau_y^6}{(\tau_\varepsilon + \tau_y)^3 (\tau_+ + \tau_y) (\tau_\chi + \tau_y)^3} \right), \tag{S2.6}$$

$$M_{0,3} = -2\pi A_- \tau_- \left(\frac{\eta_-^5 \tau_-^5}{(\tau_\varepsilon + \eta_- \tau_-)^3 (\tau_\chi + \eta_- \tau_-)^3} - \frac{\tau_+^6}{(\tau_\varepsilon + \tau_+)^3 (\tau_+ + \tau_y) (\tau_\chi + \tau_+)^3} \right), \tag{S2.7}$$

$$\begin{aligned} M_{2,1} = \frac{\pi}{2} A_- \tau_- \Bigg(& \frac{13\tau_+^3 \tau_\iota^3 + \tau_\varepsilon^4 (\tau_\iota + \tau_+)^2 + \tau_\varepsilon^3 (\tau_\iota + \tau_+)(2\tau_\iota + \tau_+)(\tau_\iota + 3\tau_+)}{(\tau_\varepsilon + \tau_\iota)(\tau_\varepsilon + 2\tau_\iota)^2 (\tau_\varepsilon + \tau_+)^2 (\tau_\chi + \tau_+)^2} - \frac{(\tau_\varepsilon + \tau_\iota)^3}{\tau_\varepsilon (\tau_\varepsilon + 2\tau_\iota)^3 (\tau_\varepsilon + \eta_- \tau_-)} \\
& + \frac{\tau_+ \tau_\varepsilon^2 \tau_\iota (3\tau_\iota + 4\tau_+)^2 + 2\tau_\varepsilon \tau_\iota^2 \tau_+^2 (8\tau_\iota + 13\tau_+)}{(\tau_\varepsilon + \tau_\iota)(\tau_\varepsilon + 2\tau_\iota)^2 (\tau_\varepsilon + \tau_+)^2 (\tau_\chi + \tau_+)^2} + \frac{(\tau_+^2 + \tau_y^2) \tau_\varepsilon^2 \tau_\chi^2 (2\tau_\varepsilon^2 + 9\tau_\varepsilon \tau_\iota + 9\tau_\iota^2)}{(\tau_\varepsilon + 2\tau_\iota)^2 (\tau_\varepsilon + \tau_+)(\tau_\chi + \tau_+)(\tau_\varepsilon + \tau_y)^2 (\tau_+ + \tau_y)(\tau_\chi + \tau_y)^2} \\
& + \frac{4\tau_+^2 \tau_y^4 (\tau_\varepsilon^2 + 3\tau_\varepsilon \tau_\iota + 2\tau_\iota^2)^2 + \tau_y^2 \tau_\varepsilon (\tau_\varepsilon^3 + 10\tau_\varepsilon^2 \tau_\iota + 24\tau_\varepsilon \tau_\iota^2 + 16\tau_\iota^3)}{(\tau_\varepsilon + \tau_\iota)^2 (\tau_\varepsilon + 2\tau_\iota)^2 (\tau_\varepsilon + \tau_+)(\tau_\chi + \tau_+)(\tau_\varepsilon + \tau_y)^2 (\tau_+ + \tau_y)(\tau_\chi + \tau_y)^2} \\
& + \frac{\tau_\iota^3}{\tau_\varepsilon (\tau_\varepsilon + 2\tau_\iota)^2 (\tau_\chi + \eta_- \tau_-)} + \frac{3\tau_+ \tau_y \tau_\varepsilon^2 \tau_\chi^2 + (\tau_+ + \tau_y) \tau_\varepsilon^2 \tau_\upsilon \tau_\chi^2}{(\tau_\varepsilon + \tau_+)(\tau_\chi + \tau_+)(\tau_\varepsilon + \tau_y)^2 (\tau_+ + \tau_y)(\tau_\chi + \tau_y)^2} \\
& + \frac{3\tau_+ \tau_y^2 \tau_\varepsilon^2 \tau_\chi (\tau_\varepsilon^2 + 5\tau_\varepsilon \tau_\iota + 5\tau_\iota^2)}{(\tau_\varepsilon + \tau_\iota)(\tau_\varepsilon + 2\tau_\iota)(\tau_\varepsilon + \tau_+)(\tau_\chi + \tau_+)(\tau_\varepsilon + \tau_y)^2 (\tau_+ + \tau_y)(\tau_\chi + \tau_y)^2} \\
& + \frac{\tau_y \tau_\varepsilon \tau_\chi + \tau_+^2 \tau_y \tau_\varepsilon^2 \tau_\chi (2\tau_\varepsilon^3 + 11\tau_\varepsilon^2 \tau_\iota + 21\tau_\varepsilon \tau_\iota^2 + 14\tau_\iota^3) + \tau_y^4 \tau_\varepsilon \tau_\chi (3\tau_\varepsilon^3 + 16\tau_\varepsilon^2 \tau_\iota + 26\tau_\varepsilon \tau_\iota^2 + 13\tau_\iota^3)}{(\tau_\varepsilon + \tau_\iota)(\tau_\varepsilon + 2\tau_\iota)^2 (\tau_\varepsilon + \tau_+)(\tau_\chi + \tau_+)(\tau_\varepsilon + \tau_y)^2 (\tau_+ + \tau_y)(\tau_\chi + \tau_y)^2} \\
& + \frac{\tau_+ \tau_y^4 \tau_\varepsilon (3\tau_\varepsilon^3 + 17\tau_\varepsilon^2 \tau_\iota + 28\tau_\varepsilon \tau_\iota^2 + 14\tau_\iota^3) + \tau_+ \tau_y^3 \tau_\varepsilon^2 (\tau_\varepsilon^3 + 12\tau_\varepsilon^2 \tau_\iota + 30\tau_\varepsilon \tau_\iota^2 + 20\tau_\iota^3)}{(\tau_\varepsilon + \tau_\iota)^2 (\tau_\varepsilon + 2\tau_\iota)(\tau_\varepsilon + \tau_+)(\tau_\chi + \tau_+)(\tau_\varepsilon + \tau_y)^2 (\tau_+ + \tau_y)(\tau_\chi + \tau_y)^2} \\
& + \frac{\tau_+^2 \tau_y^2 \tau_\varepsilon^2 (\tau_\varepsilon^4 + 11\tau_\varepsilon^3 \tau_\iota + 38\tau_\varepsilon^2 \tau_\iota^2 + 54\tau_\varepsilon \tau_\iota^3 + 27\tau_\iota^4) + \tau_+^2 \tau_y^3 \tau_\varepsilon (3\tau_\varepsilon^4 + 23\tau_\varepsilon^3 \tau_\iota + 62\tau_\varepsilon^2 \tau_\iota^2 + 70\tau_\varepsilon \tau_\iota^3 + 28\tau_\iota^4)}{(\tau_\varepsilon + \tau_\iota)^2 (\tau_\varepsilon + 2\tau_\iota)^2 (\tau_\varepsilon + \tau_+)(\tau_\chi + \tau_+)(\tau_\varepsilon + \tau_y)^2 (\tau_+ + \tau_y)(\tau_\chi + \tau_y)^2} \Bigg), \end{aligned} \tag{S2.8}$$

and

$$
\begin{aligned}
M_{1,2} = -\frac{\pi}{2} A_{-} \tau_{-} \Bigg(& \frac{13\eta_{-}^{3}\tau_{-}^{3}\tau_{\iota}^{3} + \tau_{\varepsilon}^{4}(\tau_{\iota}+\eta_{-}\tau_{-})^{2} + \tau_{\varepsilon}^{3}(\tau_{\iota}+\eta_{-}\tau_{-})(2\tau_{\iota}+\eta_{-}\tau_{-})(\tau_{\iota}+3\eta_{-}\tau_{-})}{(\tau_{\varepsilon}+\tau_{\iota})(\tau_{\varepsilon}+2\tau_{\iota})^{2}(\tau_{\varepsilon}+\eta_{-}\tau_{-})^{2}\left(\tau_{\chi}+\eta_{-}\tau_{-}\right)^{2}} \\
&+ \frac{\eta_{-}\tau_{-}\tau_{\varepsilon}^{2}\tau_{\iota}(3\tau_{\iota}+4\eta_{-}\tau_{-})^{2} + 2\eta_{-}^{2}\tau_{\varepsilon}\tau_{\iota}^{2}\tau_{-}^{2}(8\tau_{\iota}+13\eta_{-}\tau_{-})}{(\tau_{\varepsilon}+\tau_{\iota})(\tau_{\varepsilon}+2\tau_{\iota})^{2}(\tau_{\varepsilon}+\eta_{-}\tau_{-})^{2}\left(\tau_{\chi}+\eta_{-}\tau_{-}\right)^{2}} - \frac{4\tau_{+}^{4}\tau_{y}^{2} + \tau_{y}\tau_{\varepsilon}^{2}\tau_{\upsilon}\tau_{\chi}^{2} + 6\tau_{+}\tau_{y}\tau_{\varepsilon}\tau_{\upsilon}\tau_{\chi}^{2} + 7\tau_{+}\tau_{y}^{2}\tau_{\upsilon}\tau_{\chi}^{2}}{(\tau_{\varepsilon}+\tau_{+})^{2}\left(\tau_{\chi}+\tau_{+}\right)^{2}(\tau_{\varepsilon}+\tau_{y})(\tau_{+}+\tau_{y})\left(\tau_{\chi}+\tau_{y}\right)} \\
&+ \frac{7\tau_{+}\tau_{y}^{2}\tau_{\varepsilon}^{2}\tau_{\chi}^{2}}{(\tau_{\varepsilon}+\tau_{+})^{2}\left(\tau_{\chi}+\tau_{+}\right)^{2}(\tau_{\varepsilon}+\tau_{y})(\tau_{+}+\tau_{y})\left(\tau_{\chi}+\tau_{y}\right)\left(\tau_{\varepsilon}+2\tau_{\iota}\right)} - \frac{(\tau_{\varepsilon}+\tau_{\iota})^{3}}{\tau_{\varepsilon}(\tau_{\varepsilon}+2\tau_{\iota})^{3}(\tau_{\varepsilon}+\tau_{+})} \\
&+ \frac{\tau_{+}^{2}\tau_{\varepsilon}^{2}\tau_{\chi}^{2}(\tau_{\varepsilon}+3\tau_{\iota})(2\tau_{\varepsilon}+3\tau_{\iota}) + \tau_{y}^{2}\tau_{\varepsilon}^{2}\tau_{\chi}^{2}(\tau_{\varepsilon}^{2}+3\tau_{\varepsilon}\tau_{\iota}+3\tau_{\iota}^{2}) + \tau_{+}^{4}\tau_{\varepsilon}\tau_{\chi}(3\tau_{\varepsilon}^{2}+13\tau_{\varepsilon}\tau_{\iota}+13\tau_{\iota}^{2})}{(\tau_{\varepsilon}+2\tau_{\iota})^{2}(\tau_{\varepsilon}+\tau_{+})^{2}\left(\tau_{\chi}+\tau_{+}\right)^{2}(\tau_{\varepsilon}+\tau_{y})(\tau_{+}+\tau_{y})\left(\tau_{\chi}+\tau_{y}\right)} \\
&+ \frac{(\tau_{+}^{4}\tau_{y}\tau_{\varepsilon}+\tau_{+}^{3}\tau_{y}^{2}\tau_{\varepsilon}^{2})(3\tau_{\varepsilon}^{2}+14\tau_{\varepsilon}\tau_{\iota}+14\tau_{\iota}^{2}) + 3\tau_{+}^{2}\tau_{y}\tau_{\varepsilon}^{2}\tau_{\chi}(\tau_{\varepsilon}^{2}+5\tau_{\varepsilon}\tau_{\iota}+5\tau_{\iota}^{2}) + \tau_{+}\tau_{\varepsilon}^{4}\tau_{\chi}(\tau_{\iota}+\tau_{y})(\tau_{\iota}+2\tau_{y})}{(\tau_{\varepsilon}+\tau_{\iota})(\tau_{\varepsilon}+2\tau_{\iota})(\tau_{\varepsilon}+\tau_{+})^{2}\left(\tau_{\chi}+\tau_{+}\right)^{2}(\tau_{\varepsilon}+\tau_{y})(\tau_{+}+\tau_{y})\left(\tau_{\chi}+\tau_{y}\right)} \\
&+ \frac{\tau_{\iota}^{3}}{\tau_{\varepsilon}(\tau_{\varepsilon}+2\tau_{\iota})^{2}\left(\tau_{\chi}+\tau_{+}\right)} + \frac{\tau_{+}^{3}\tau_{\varepsilon}^{2}\tau_{\chi}}{(\tau_{\varepsilon}+\tau_{\iota})(\tau_{\varepsilon}+2\tau_{\iota})(\tau_{\varepsilon}+\tau_{+})^{2}\left(\tau_{\chi}+\tau_{+}\right)^{2}(\tau_{\varepsilon}+\tau_{y})(\tau_{+}+\tau_{y})\left(\tau_{\chi}+\tau_{y}\right)} \\
&+ \frac{\tau_{+}^{2}\tau_{y}^{2}\tau_{\varepsilon}^{2}(\tau_{\varepsilon}^{4}+11\tau_{\varepsilon}^{3}\tau_{\iota}+38\tau_{\varepsilon}^{2}\tau_{\iota}^{2}+54\tau_{\varepsilon}\tau_{\iota}^{3}+27\tau_{\iota}^{4})}{(\tau_{\varepsilon}+\tau_{\iota})^{2}(\tau_{\varepsilon}+2\tau_{\iota})^{2}(\tau_{\varepsilon}+\tau_{+})^{2}\left(\tau_{\chi}+\tau_{+}\right)^{2}(\tau_{\varepsilon}+\tau_{y})(\tau_{+}+\tau_{y})\left(\tau_{\chi}+\tau_{y}\right)} \\
&+ \frac{\tau_{+}^{3}\tau_{y}\tau_{\varepsilon}^{2}(\tau_{\varepsilon}^{2}+10\tau_{\varepsilon}\tau_{\iota}+10\tau_{\iota}^{2})}{(\tau_{\varepsilon}+\tau_{\iota})^{2}(\tau_{\varepsilon}+\tau_{+})^{2}\left(\tau_{\chi}+\tau_{+}\right)^{2}(\tau_{\varepsilon}+\tau_{y})(\tau_{+}+\tau_{y})\left(\tau_{\chi}+\tau_{y}\right)} \Bigg).
\end{aligned}
\tag{S2.9}
$$
